# Computational Identification of Genomic Features That Influence 3D Chromatin Domain Formation

**DOI:** 10.1371/journal.pcbi.1004908

**Published:** 2016-05-20

**Authors:** Raphaël Mourad, Olivier Cuvier

**Affiliations:** Laboratoire de Biologie Moléculaire Eucaryote (LBME), CNRS, Université Paul Sabatier (UPS), Toulouse, France; University of Pennsylvania, UNITED STATES

## Abstract

Recent advances in long-range Hi-C contact mapping have revealed the importance of the 3D structure of chromosomes in gene expression. A current challenge is to identify the key molecular drivers of this 3D structure. Several genomic features, such as architectural proteins and functional elements, were shown to be enriched at topological domain borders using classical enrichment tests. Here we propose multiple logistic regression to identify those genomic features that positively or negatively influence domain border establishment or maintenance. The model is flexible, and can account for statistical interactions among multiple genomic features. Using both simulated and real data, we show that our model outperforms enrichment test and non-parametric models, such as random forests, for the identification of genomic features that influence domain borders. Using *Drosophila* Hi-C data at a very high resolution of 1 kb, our model suggests that, among architectural proteins, BEAF-32 and CP190 are the main positive drivers of 3D domain borders. In humans, our model identifies well-known architectural proteins CTCF and cohesin, as well as ZNF143 and Polycomb group proteins as positive drivers of domain borders. The model also reveals the existence of several negative drivers that counteract the presence of domain borders including P300, RXRA, BCL11A and ELK1.

## Author Summary

Chromosomal DNA is tightly packed up in 3D such that around 2 meters of this long molecule fits into the microscopic nucleus of every cell. The genome packing is not random, but instead structured in 3D domains that are essential to numerous key processes in the cell, such as for the regulation of gene expression or for the replication of DNA. A current challenge is to identify the key molecular drivers of this higher-order chromosome organization. Here we propose a novel computational integrative approach to identify proteins and DNA elements that positively or negatively influence the establishment or maintenance of 3D domains. Analysis of *Drosophila* data at very high resolution suggests that among architectural proteins, BEAF-32 and CP190 are the main positive drivers of 3D domains. In humans, our results highlight the roles of CTCF, cohesin, ZNF143 and Polycomb group proteins as positive drivers of 3D domains, in contrast to P300, RXRA, BCL11A and ELK1 that act as negative drivers.

## Introduction

High-throughput chromatin conformation capture (Hi-C) has emerged over the past years as an efficient approach to map long-range chromatin contacts [1–3]. This technique has allowed the study of the 3D architecture of chromosomes at an unprecedented resolution for many genomes and cell types [4–7]. Multiple hierarchical levels of genome organization have been revealed: compartments A/B [1], sub-compartments [8], topologically associating domains (TADs) [4, 5] and sub-TADs [7]. Among those domains, TADs represent a pervasive structural feature of the genome organization. TADs are stable across different cell types and highly conserved across species.

A current challenge is to identify the molecular drivers of topological arrangements of higher-order chromatin organization. There is a growing body of evidence that insulator binding proteins (IBPs) such as CTCF, and cofactors such as cohesin, act as mediators of long-range chromatin contacts [5, 6, 9–11]. In human, depletion of cohesin predominantly reduces interactions within TADs, whereas depletion of CTCF not only decreases intradomain contacts but also increases interdomain contacts [12]. The densest Hi-C mapping in human has recently revealed that loops that demarcate domains are often marked by asymmetric CTCF motifs where cohesin is recruited [8]. In *Drosophila*, silencing of cohesin and condensin II have recently demonstrated their roles on long-range contacts [13]. In addition, numerous IBPs, cofactors and functional elements colocalize at TAD borders [11]. However it is unclear if all these proteins and functional elements, or specific combinations of them, play a role in TAD border establishment or maintenance. Computational approaches that integrate protein binding (chromatin immunoprecipitation followed by high-throughput DNA sequencing, ChIP-seq) with Hi-C data may be well-suited to identify the key drivers of chromatin architecture.

Most computational approaches dedicated to chromosome conformation analysis have focused on correcting contact matrices for experimental biases [6, 14–16] in order to assess more precisely the significance of contact counts [17, 18], to identify chromatin compartments [1, 15, 19], or to 3D model chromosome folding [1, 5, 20–22]. However few computational methods have been proposed to study the roles of DNA-binding proteins and functional elements in chromosome folding. A simple yet widely used statistical method consists in assessing enrichment of a genomic feature around 3D domain borders by Fisher’s exact or Pearson’s chi-squared tests [4, 5, 7]. An important caveat of enrichment test is that it only identifies those genomic features that colocalize at domain borders, but it cannot determine which genomic features influence the domain border establishment or maintenance. For instance, two genomic features might be both found significantly enriched at domain boundaries, but only one of them might truly influence the domain border establishment or maintenance. This is due to the colocalization (correlation) between the two genomic features. Statistically speaking, correlation does not imply causation. Other works focused on the prediction of 3D domain borders using (semi) non-parametric models and identified a subset of genomic features that are the most predictive of TADs [23, 24]. However a genomic feature can efficiently predict 3D domain borders without being influential [25].

In this paper, we propose a multiple logistic regression to assess the influence of genomic features such as DNA-binding proteins and functional elements on topological chromatin domain borders. Compared to enrichment test and non-parametric models, multiple logistic regression assesses conditional independence and thus can identify most influential proteins with respect to domain borders. Moreover the multiple logistic regression model can easily accommodate interactions between genomic features to assess the impact of co-occurences on domain borders. We illustrate our model using recent *Drosophila* and human Hi-C data allowing to probe TAD borders depending on multiple proteins and functional elements. Using both simulated and real data, we show that our model outperforms enrichment test and non-parametric models such as random forests for the identification of known and suspected architectural proteins. In addition, the proposed method identifies genomic features that positively or negatively impact TAD borders with a very high resolution of 1 kb.

## Results

### The model

The proposed multiple logistic regression models the influences of *p* genomic features on 3D domain borders:

$$\text{ln}\frac{Prob(Y=1|X)}{1-Prob(Y=1|X)}=\beta_{0}+\beta X$$ (1)

Where **X** = {*X*_1_, …, *X*_*p*_} is the set of *p* genomic features such as DNA-binding proteins and *Y* is a variable that indicates if the genomic bin belongs to a border (*Y* = 1) or not (*Y* = 0). The set ***β*** = {*β*_1_, …, *β*_*p*_} denotes slope parameters, one parameter for each genomic feature. The model can easily accommodate interaction terms between genomic features (see Subsection Materials and Methods, Analysis of interactions). By default, model likelihood is maximized by iteratively reweighted least squares to estimate unbiaised parameters. However, when there are a large number of correlated genomic features in the model, L1-regularization is used instead to reduce instability in parameter estimation [26].

**Fig 1:**
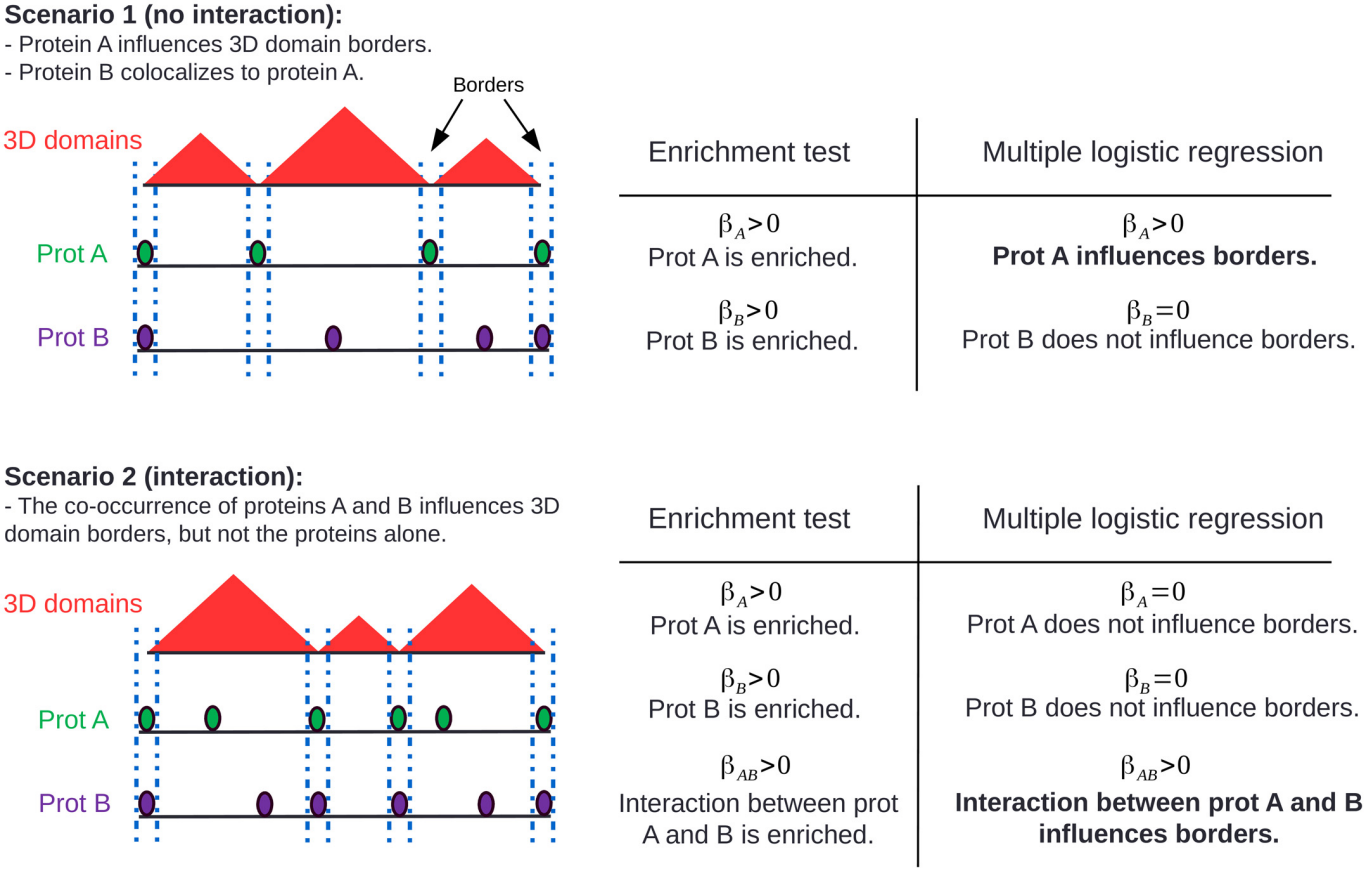
Illustration of the proposed multiple logistic regression to assess the influences of genomic features on 3D domain borders and comparison with enrichment test.

We illustrate the proposed model using two scenarios and compare it with enrichment test (Fig 1). In the first scenario, protein A positively influences 3D domain borders, while protein B colocalizes to protein A. In this scenario, enrichment test will estimate that the parameter associated with protein A *β*_*A*_ > 0 and the parameter associated with protein B *β*_*B*_ > 0. In other words, both proteins A and B are enriched at 3D domain borders. Multiple logistic regression will instead estimate that parameters *β*_*A*_ > 0 and *β*_*B*_ = 0. This means that protein A positively influences 3D domain borders, while protein B does not. This is because multiple logistic regression can discard spurious associations (here between protein B and 3D domain borders). One would argue that enrichment test can also be used to discard the spurious association if the enrichment of protein B when protein A is absent is tested instead. However such conditional enrichment test becomes intractable when more than 3 proteins colocalize to domain borders, whereas multiple logistic regression is not limited by the numbers of proteins to analyze within the same model.

In the second scenario, the co-occurrence of proteins A and B influences 3D domain borders, but not the proteins alone. Enrichment test will find that each protein alone is enriched at 3D domain borders (*β*_*A*_ > 0 and *β*_*B*_ > 0) as well as their interaction (*β*_*AB*_ > 0). The proposed model will instead find that only the interaction between proteins A and B influences 3D domain borders (*β*_*A*_ = 0, *β*_*B*_ = 0 and *β*_*AB*_ > 0).

In addition to these two previous scenarios, another interest of the model is the possibility to study the negative influence of a protein (or of a co-occurence of proteins) on TAD border establishment of maintenance. In other words, its presence counteracts the establishment or maintenance of 3D domain borders. In such scenario, multiple logistic regression will estimate a parameter *β* < 0 (see below).

Depending on the parameter estimation algorithm used (likelihood maximization or L1-regularization), results are interpreted differently. If likelihood maximization is used, then a protein beta parameter can be considered as significantly different from zero if the corresponding p-value is lower than the significance level computed by Bonferroni procedure. If L1-regularization is used instead, then p-values are not computed. A protein is considered as influential if its beta parameter is different from zero. Using both algorithms, the beta parameter is the only measure used to quantify how strong is the influence of a protein on the 3D domain borders, and the p-value should not be used instead because it depends on the amount of data available. Both algorithms are useful in practice. Likelihood maximization allows to estimate beta parameters without any bias but influential proteins should be known in advance. L1-regularization can be useful to select the influential proteins among a large set of correlated candidates, but estimates will be biased.

### Parameter estimation accuracy

Several characteristics of the analyzed ChIP-seq and functional element data might prevent the accurate estimation of multiple logistic regression parameters ***β***. The matrix **X** of genomic features is sparse (numerous values equal zero) because genomic features are often absent from a particular genomic bin. Sparsity of matrix **X** is known to prevent convergence of likelihood maximization for parameter estimation [27]. Moreover some genomic features can be correlated. For instance, different insulator binding proteins might bind to the same genomic regions. For all these reasons, accurate estimation of parameters could fail in theory. Hence we evaluated the accuracy of parameter estimation using simulations.

**Fig 2:**
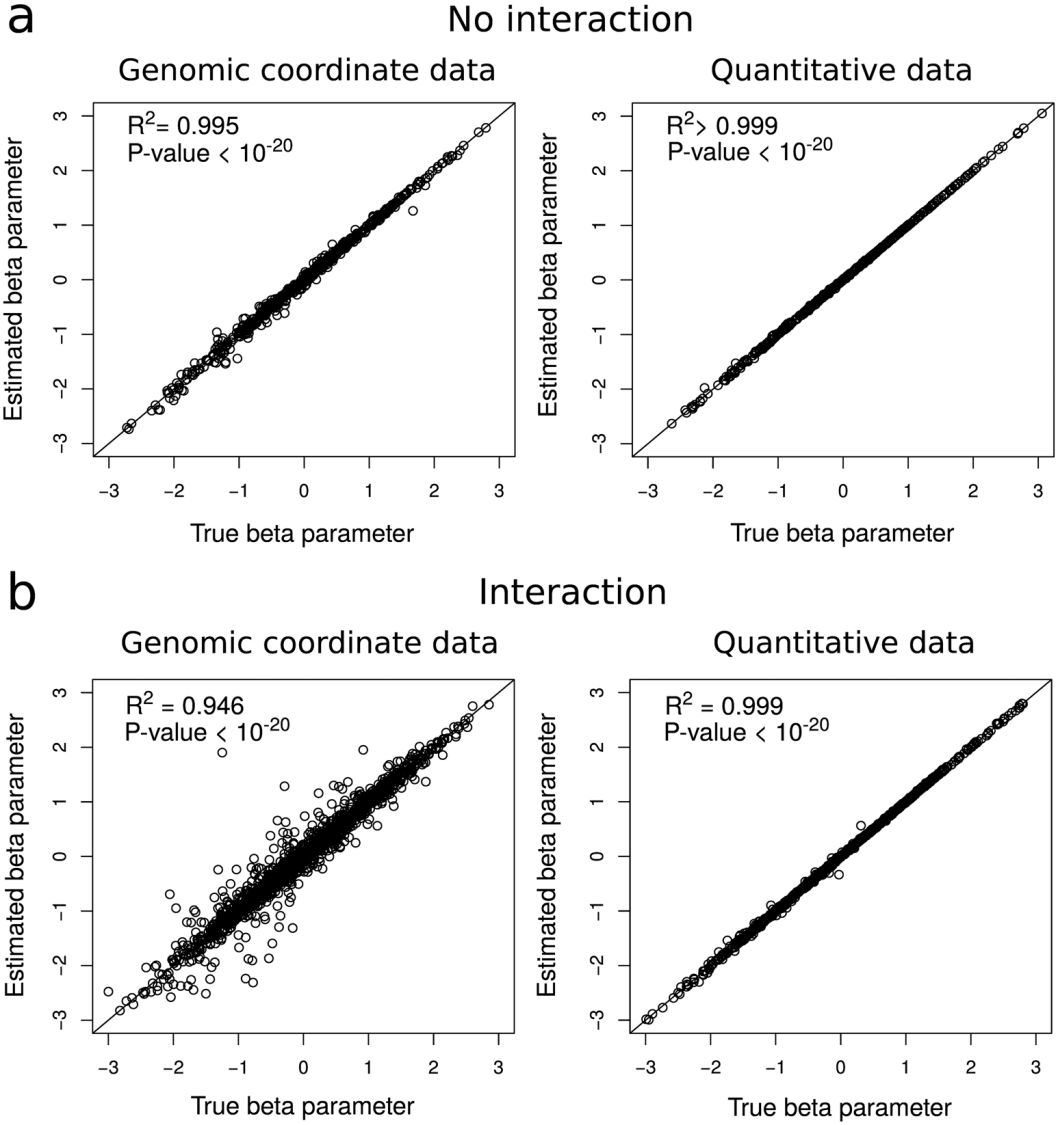
Parameter estimation accuracy of multiple logistic regression. a) Estimated versus true parameter for marginal genomic features (the model does not include any interaction between genomic features). b) Estimated versus true parameter for two-way interactions between genomic features (*i.e.* for any interaction between two genomic features, see Subsection Materials and Methods, Analysis of interactions). Genomic coordinate data are ChIP-seq peak coordinates. Quantitative data are ChIP-seq signal intensities $\text{log}\frac{ChIP}{Input}$.

We simulated data that were similar to real ChIP-seq data (see Subsection Materials and Methods, Data simulation, first paragraph). Both genomic coordinate data (*e.g.*, ChIP-seq peak coordinates) and quantitative data (*e.g.*, ChIP-seq signal intensity $\text{log}\frac{ChIP}{Input}$) were generated. From the simulated data, multiple logistic regression model parameters were then estimated by maximum likelihood. We first simulated 100 genomic coordinate and 100 quantitative datasets that comprised 6 proteins and learned models without considering any interaction terms. In Fig 2a, we plotted true against estimated parameter values. We reported a very good accuracy for parameter estimation for both genomic coordinate and quantitative data with *R*^2^ = 99.5% (*p* < 1 × 10^−20^) and *R*^2^ > 99.9% (*p* < 1 × 10^−20^) between true and estimated parameter values, respectively. Because some proteins might be rare over the genome and only involved in some 3D domain borders, we studied parameter accuracy for simulated proteins with varied ChIP-seq peak numbers. Parameter estimation was highly accurate even for proteins with a low number of peaks over the genome (*R*^2^ = 97.4% for 50 peaks; S1 Fig). In addition, we sought to assess how parameter estimation is affected by 3D domain border inaccuracy of few kilobases. We observed that with a border inaccuracy equal or lower than 2 kb, parameter estimation was still accurate (*R*^2^ > 70.9%, S2 Fig). We then simulated 100 genomic coordinate and 100 quantitative datasets that comprised the same 6 proteins and learned models with all two-way (*e.g.* *X*_1_ *X*_2_) interaction terms. In Fig 2b, we plotted true against estimated parameter values corresponding to interaction terms only. Parameter estimation accuracy was still high for both genomic coordinate data (*R*^2^ = 94.6%, *p* < 1 × 10^−20^) and quantitative data (*R*^2^ = 99.9%, *p* < 1 × 10^−20^). We concluded that model parameter estimation was accurate for both marginal and two-way interaction of genomic features.

### MLR outperforms enrichment test and random forests to identify drivers of TAD borders

**Fig 3:**
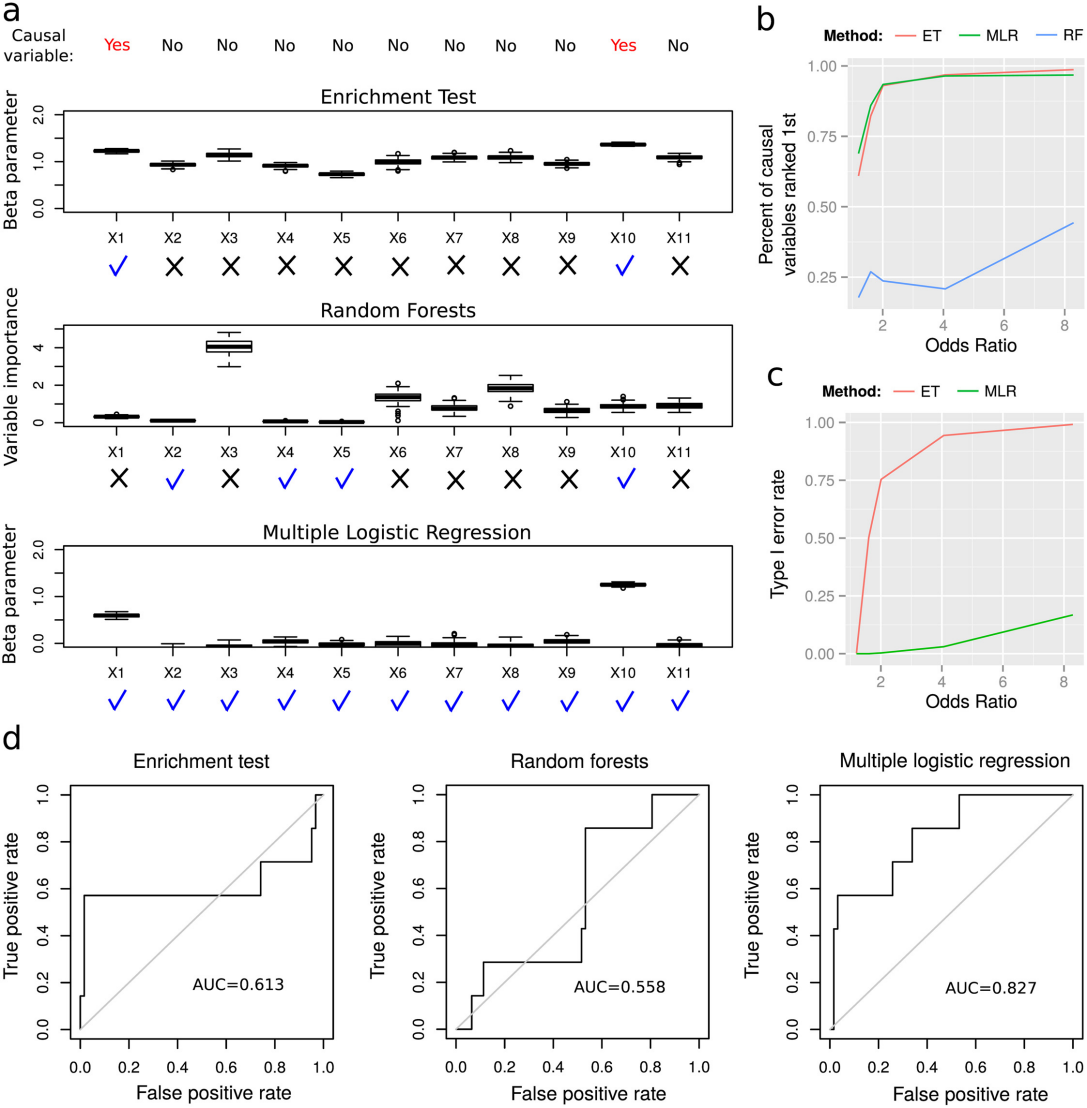
Comparisons between multiple logistic regression (MLR), enrichment test (ET) and random forests (RF) on simulated and real data. a) Comparison of MLR beta parameters with ET beta parameters and RF variable importances obtained from 100 simulated datasets including 11 genomic features. Among the genomic features, variables *X*_1_ and *X*_10_ were chosen to be causal. For a method, a blue check mark denotes a causal or non-causal variable that was correctly identified as causal (resp. non-causal). A black x mark denotes a causal or non-causal variable that was incorrectly identified as non-causal (resp. causal). b) Percents of causal variables ranked first by ET, MLR and RF computed from 100 simulated datasets and varying odds ratios. Here the causal variables and their number were randomly drawn at each simulation. c) Type I error rates for MLR and ET computed from 100 simulated datasets. RF were not included because no p-values were available. The significance threshold *α* was set to 10^−5^. Simulated data were the same as in b). d) Comparison of MLR with ET and RF to detect known or suspected architectural proteins in human using GM12878 cell ChIP-seq data. Receiver operating characteristic (ROC) curves were computed from Wald’s statistics for ET, from beta parameters for MLR, and from variable importances for random forests. Computations were carried out at 1 kb resolution.

We then sought to assess how multiple logistic regression (MLR) efficiently identifies genomic features that influence TAD borders, comparing with other approaches commonly used to assess the link between TAD borders and genomic features. We compared our model with enrichment test (ET) [4] and non-parametric model [23]. For the non-parametric model, we used random forests (RF) which are very similar to the model used in [23], but for which a scalable implementation allowed high resolution analysis (https://github.com/aloysius-lim/bigrf). For this purpose, we first simulated 100 datasets comprising 11 genomic features {*X*_1_, *X*_2_, …, *X*_11_} that were similar to real ChIP-seq data (see Subsection Materials and Methods, Data simulation, second paragraph). Among the genomic features, variables *X*_1_ and *X*_10_ were chosen to be causal with an odds ratio of 4, which was comparable to odds ratios estimated from real data (see below). We compared beta parameters from multiple logistic regression with beta parameters from enrichment test and variable importances from random forests (Fig 3a). Enrichment test correctly identified causal variables *X*_1_ and *X*_10_ as the most enriched (beta median = 1.3), but also found highly enriched non-causal variables (beta median = 1). Random forests detected *X*_3_ and *X*_8_ as the most influential variables for prediction (variable importance median >2.75), although they were not causal genomic features. In contrast, multiple logistic regression correctly identified *X*_1_ and *X*_10_ as influential variables (beta median = 0.93) and discarded non-causal variables (beta median = −0.03).

We next simulated more complex scenarios for which the causal variables and their number were randomly chosen for each simulation. In addition, simulations were carried out for different odds ratios to study the influence of effect size. As previously, we compared multiple logistic regression with enrichment test and random forests. For each method, we computed the percentage of models that correctly ranked first the causal variables in terms of beta parameter or variable importance (Fig 3b). We observed that both enrichment test and multiple logistic regression successfully ranked first the causal variables even for a low odds ratio of 2 (93% of models), whereas random forests mostly failed even for the easiest scenario (44% of models for an odds ratio of 8; in the next paragraph, we will see that random forests poorly performed here partly due to high data sparsity). We then compared empirical type I error rate for a significance threshold *α* = 10^−5^ between enrichment test and multiple logistic regression for which p-values on beta coefficients were available (Fig 3c). Even for a high odds ratio of 8, MLR had a low error rate of 16%. Conversely enrichment test showed a high error rate of 75% even for an odds ratio of 2.

We also compared MLR with ET and RF using real data in human. For this purpose, we analyzed new 3D domains detected from recent high resolution Hi-C data at 1 kb for GM12878 cells for which 69 ChIP-seq data were available [8]. Multiple lines of evidence indicate that CTCF and cohesin serve as mediators of long-range contacts [5, 6, 9–11, 28]. However several proteins also colocalize or interact with CTCF, including Yin Yang 1 (YY1), Kaiso, MYC-associated zing-finger protein (MAZ), jun-D proto-oncogene (JUND) and ZNF143 [29]. In addition, recent work has demonstrated the spatial clustering of Polycomb repressive complex proteins [30]. Using the large number of available proteins in GM12878 cells, we could compare MLR with ET and RF to identify known or suspected architectural proteins CTCF, cohesin, YY1, Kaiso, MAZ, JUND, ZNF143 and EZH2. For this purpose, we computed receiver operating characteristic (ROC) curves using Wald’s statistics for ET, beta parameters for MLR, and variable importances for RF. We carried out computations at the very high resolution of 1 kb (see Subsection Materials and Methods, Binned data matrix). ROC curves revealed that MLR clearly outperformed ET and RF to identify architectural proteins (*AUC*_*MLR*_ = 0.827; Fig 3d). Lower performance of ET (*AUC*_*ET*_ = 0.613) was likely due to its inability to account for correlations among the proteins (average correlation = 0.19). Regarding RF, its low performance (*AUC*_*RF*_ = 0.558) could be explained by its well-known inefficiency with sparse data (at 1kb, there were 99.4% of zeros in the data matrix **X**). At a lower resolution of 40 kb (88.5% of zeros), RF performed much better (*AUC*_*RF*_ = 0.746) but still lower than MLR (*AUC*_*MLR*_ = 0.815; S3 Fig).

**Fig 4:**
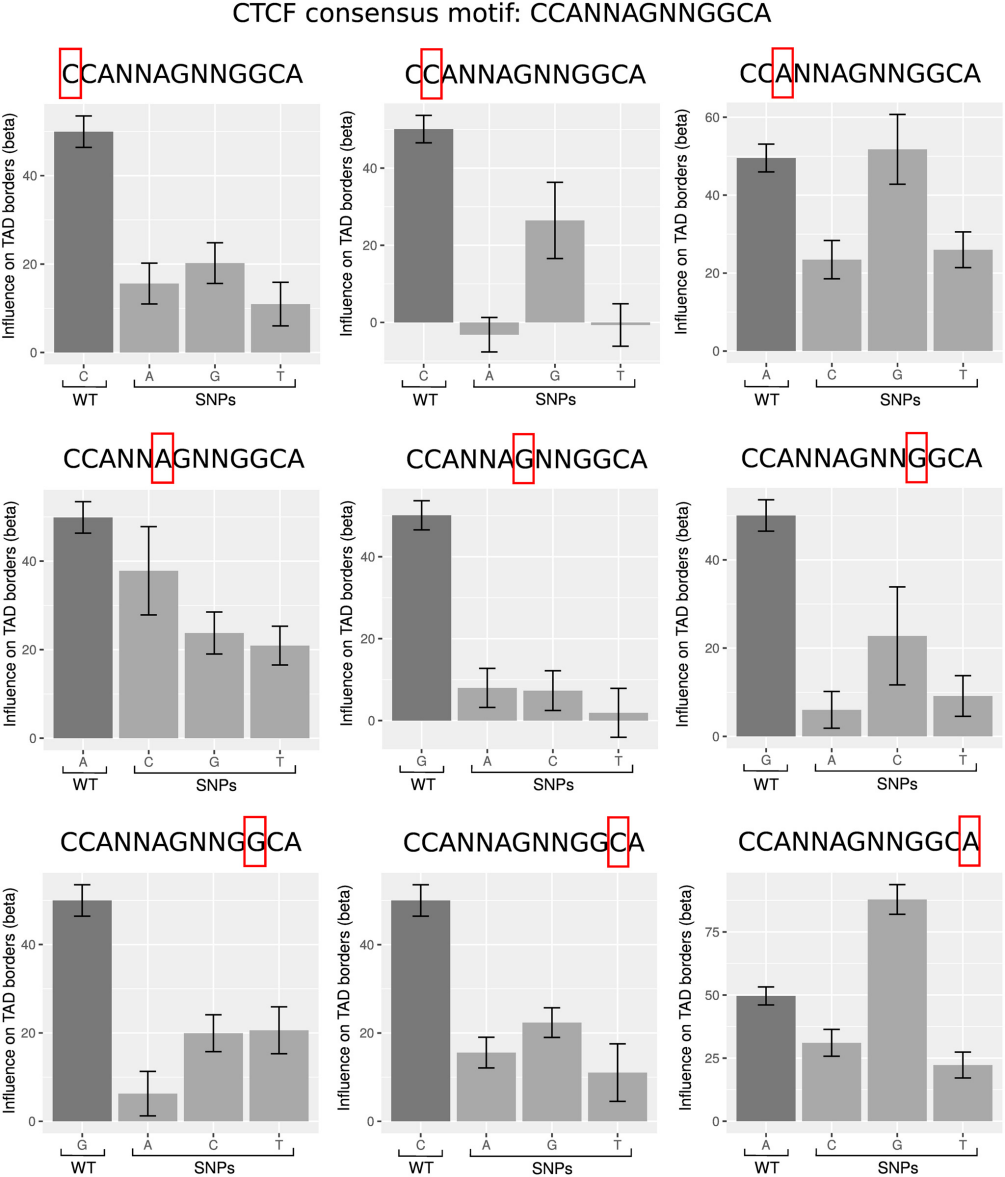
Analysis of the impacts of single nucleotide polymorphisms on the consensus CTCF motif in human GM12878 cells.

To further validate MLR results with real data, we analyzed the impacts of single nucleotide polymorphisms (SNPs) in the consensus CTCF motif in human. SNPs play an important role in common genetic diseases and recent works have uncovered differential long-range contacts due to variations in the CTCF motif [31–33]. SNPs in the consensus CTCF motif are thus expected to affect, and most likely to decrease, the influence of CTCF motif on 3D domain border establishment or maintenance. We then tested if MLR was able to detect the impacts of SNPs on CTCF motif. For this purpose, we included within the same MLR model the wild-type (WT) motif and the three alternative alleles for a given position in the motif. For instance, for the first position, the MLR comprised genomic coordinates of the WT motif CCANNAGNNGGCA and the genomic coordinates of the mutated motifs ACANNAGNNGGCA, GCANNAGNNGGCA and TCANNAGNNGGCA. Over 27 mutated CTCF motifs, 25 showed beta coefficients that were lower than the one of WT CTCF motif, indicating that the corresponding SNPs diminished the influence of CTCF motif on TAD borders as expected (Fig 4). Because correlations among the motif variables were very low (average correlation <0.01), ET performed as efficiently as MLR to detect the influences of SNPs (*AUC*_*ET*_ = 0.926 and *AUC*_*MLR*_ = 0.926), but RF was inaccurate (*AUC*_*RF*_ = 0.638; S4 Fig). For instance, for the first position, we observed that all three alternative alleles (A, G and T) diminished the influence of the motif with respect to 3D domain borders. Some mutations even canceled the influence of CTCF motif (for instance, alleles A and T on position 2). On the last position, allele G had a higher influence than the WT motif. This result was actually consistent with the ambiguity between allele A and G in the motif. Similar results were obtained for consensus BEAF-32 motif CGATA in *Drosophila* (S5 Fig).

Using both simulated and real data, we concluded that multiple logistic regression correctly identified causal variables and discarded spurious associations of non-causal variables with TAD borders while both enrichment test and random forests failed. In addition, multiple logistic regression successfully predicted expected effects of SNPs on CTCF and BEAF-32 motifs known to influence long-range contacts in human and *Drosophila*, respectively. These predicted effects of SNPs could further serve to identify new regulatory variants in the context of genome-wide association studies.

### BEAF-32 influences TAD borders in *Drosophila*

We implemented the proposed model such that it can deal with either genomic coordinate data or quantitative data. However, in the present study, we chose to focus on genomic coordinate data as in [11, 34]. An advantage of this approach was that both DNA-binding proteins and functional elements could be included within the same model. In addition, we observed that logistic regression models built from genomic coordinate data usually outperformed those obtained with quantitative data in terms of deviance ratio and AIC (model deviance ratios and AICs are given in S1 Table).

The influences of genomic features such as DNA-binding proteins or gene transcription on TAD border establishment or maintenance can be estimated by the proposed multiple logistic regression. Using *Drosophila* Kc167 cell Hi-C data at 1 kb resolution, we assessed the effects of insulator binding proteins, cofactors, gene transcription and functional elements on TAD borders. Although TADs were computed from 1 kb resolution Hi-C data, genomic features were binned at an even higher resolution of 50 bp in order to better discriminate between genomic features that influence TAD borders and those that do not, and to reduce standard errors of model parameters (see Subsection Materials and Methods, Binned data matrix). In this subsection, we first focused on the effects of insulator binding proteins in driving TAD borders [35].

**Fig 5:**
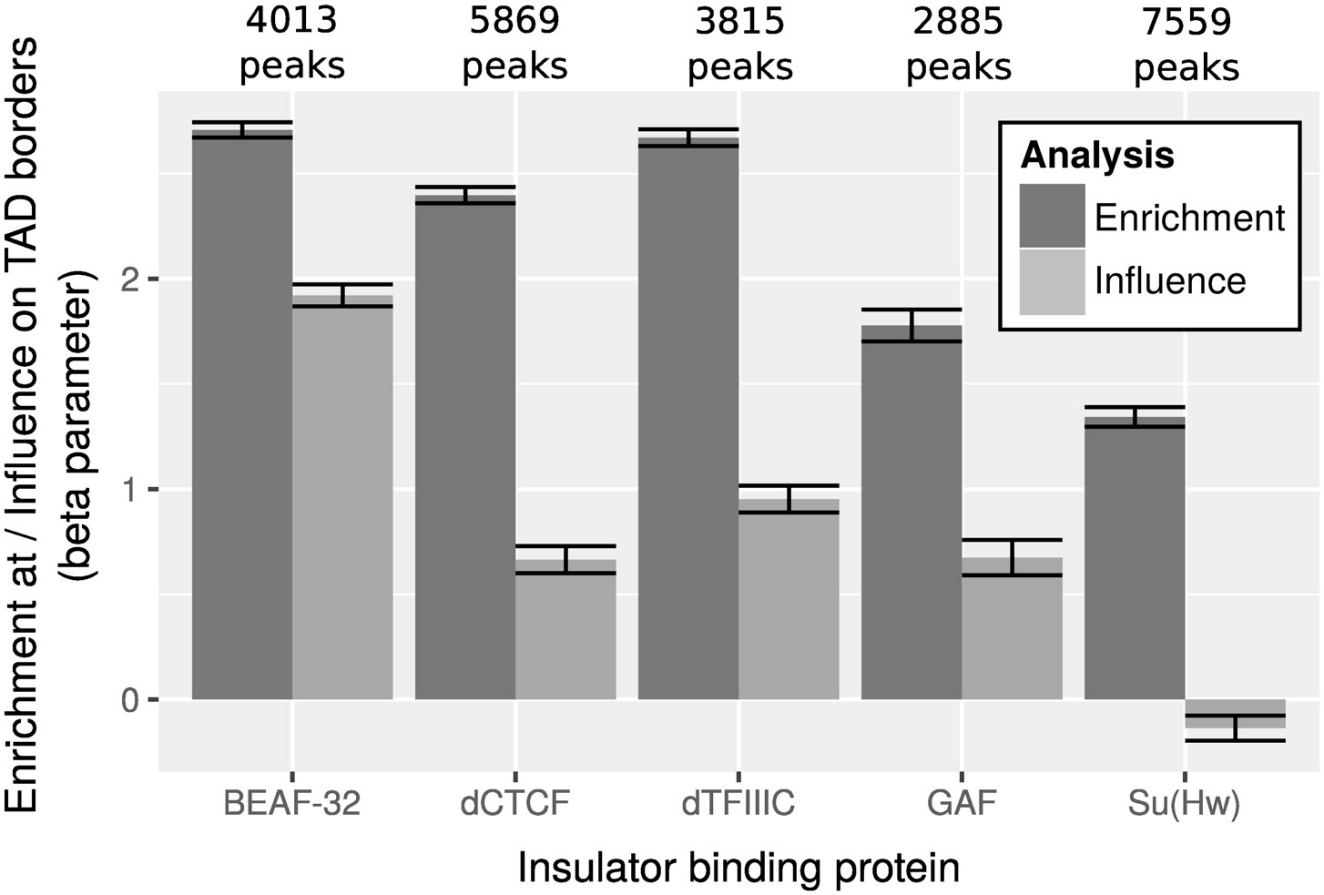
Comparison between enrichments by enrichment tests and influences by multiple logistic regression of insulator binding proteins at topologically associating domain (TAD) borders of wild-type *Drosophila* Kc167 cells. In both enrichment test and multiple logistic regression, beta parameters are computed and displayed. Error bars show 95% confidence intervals of beta parameters.

In *Drosophila*, there are five subclasses of insulator sequences [36]. Each subclass is bound by a particular type of insulator binding protein (IBP): suppressor of hairy wing (Su(Hw)), *Drosophila* CTCF (dCTCF), boundary-element-associated factor of 32 kDa (BEAF-32), GAGA binding factor (GAF), and Zeste-White 5 (ZW5) [10]. In addition, the general transcription factor dTFIIIC was recently identified as a new IBP [11]. We assessed enrichments of these IBPs within TAD borders (Fig 5). We observed enrichments for all these IBPs (all coefficients $\hat{\beta }>1.34$ and all p-values *p* < 1 × 10^−20^). BEAF-32 was the most enriched IBP with a coefficient $\hat{\beta }=2.71$, corresponding to an odds ratio $\hat{OR}=15.03$, whereas GAF was the least enriched IBP with a coefficient $\hat{\beta }=1.34$, corresponding to an odds ratio $\hat{OR}=3.82$.

Multiple logistic regression yielded different results (Fig 5). All beta coefficients decreased reflecting colocalization among the proteins (average correlation of 0.28). Despite these correlations, the tight 95% confidence intervals reflect that betas were estimated with low standard errors. This is due to the very large number of observations (>1 million) compared to the low number of variables (6 variables) obtained for a binning at 50 bp. There were clear differences of betas among the IBPs compared with enrichment analysis [5, 6]. Only BEAF-32 showed high and significant beta (BEAF-32: $\hat{\beta }=1.92$, *p* < 1 × 10^−20^). For other IBPs, betas were significant but much lower ($\hat{\beta }<0.95$, *p* < 1 × 10^−20^). Thus although dCTCF, dTFIIIC, GAF and Su(Hw) were enriched at TAD borders, multiple logistic regression revealed that they weakly influence TAD borders. High enrichments of these proteins are due to their correlations with BEAF-32. For instance, previous work showed that numerous dCTCF sites align tightly with BEAF-32 [37]. These results supported the role of BEAF-32 as most influential IBP of TAD borders.

### Architectural proteins impact more TAD-based organization than transcription

There has been an ongoing debate to know whether transcription or architectural proteins are the main cause of TAD border demarcation [6]. Using enrichment test, we observed that active transcription start sites (TSSs) were enriched at TAD borders ($\hat{\beta }=1.82$, *p* < 1 × 10^−20^), as well as architectural proteins such as BEAF-32 ($\hat{\beta }=2.72$, *p* < 1 × 10^−20^). Using multiple logistic regression, we then estimated the effects of transcription and of architectural proteins on TAD borders within the same model (S6 Fig). We observed that active TSSs had a significant positive effect in TAD border establishment/maintenance ($\hat{\beta }=0.42$, *p* < 1 × 10^−20^). This effect was much lower than the one of architectural protein BEAF-32 ($\hat{\beta }=2.59$, *p* < 1 × 10^−20^). Our model thus reveals that architectural protein BEAF-32 contributes much more to TAD-based organization than transcription. However one might argue that the comparison between active TSSs and BEAF-32 was not straightforward because the latter represented two distinct genomic features, a functional element and a protein, respectively. Hence for a proper comparison between transcription and architectural proteins, we compared within the same multiple logistic regression the effects of the short isoform of *Drosophila* Brd4 homologue (Fs(1)h-S), a major transcriptional factor involved in transcriptional activation, with the long isoform (Fs(1)h-L), a recently identified architectural protein [38]. We observed that Fs(1)h-S had a significant positive effect on TAD borders ($\hat{\beta }=1.87$, *p* < 1 × 10^−20^), but which was lower than the one of Fs(1)h-L ($\hat{\beta }=2.60$, *p* < 1 × 10^−20^). Our results thus highlighted the prevalent roles of architectural proteins compared to transcription, which was highly consistent with recent results suggesting a lower impact of transcription [13].

### The role of cofactors in *Drosophila*

Recent work supported the idea that IBPs may favor long-range contacts by recruiting cofactors directly involved in stabilizing long-range contacts [8–10]. In *Drosophila*, several cofactors were identified: condensin I, condensin II, Chromator, centrosomal protein of 190 kDa (CP190), cohesin [10, 13, 39, 40] and Fs(1)h-L [38]. We first analyzed by multiple logistic regression all abovementioned cofactors in their own to understand their relative contribution to TAD borders (S7 Fig). Among the cofactors, CP190 had the highest influence on TAD borders in agreement with previous findings [5] ($\hat{\beta }=1.12$, *p* < 1 × 10^−20^). Because cofactors were expected to be recruited by IBPs to the chromatin [8, 9, 39, 40], we then regressed cofactors with all IBPs and all IBP-cofactor interactions (see S2 Table). We observed that CP190 still presented a high beta ($\hat{\beta }=1.13$, *p* < 1 × 10^−20^), which reflect that additional IBPs are able to recruit these cofactors in concordance with recent results [41].

**Fig 6:**
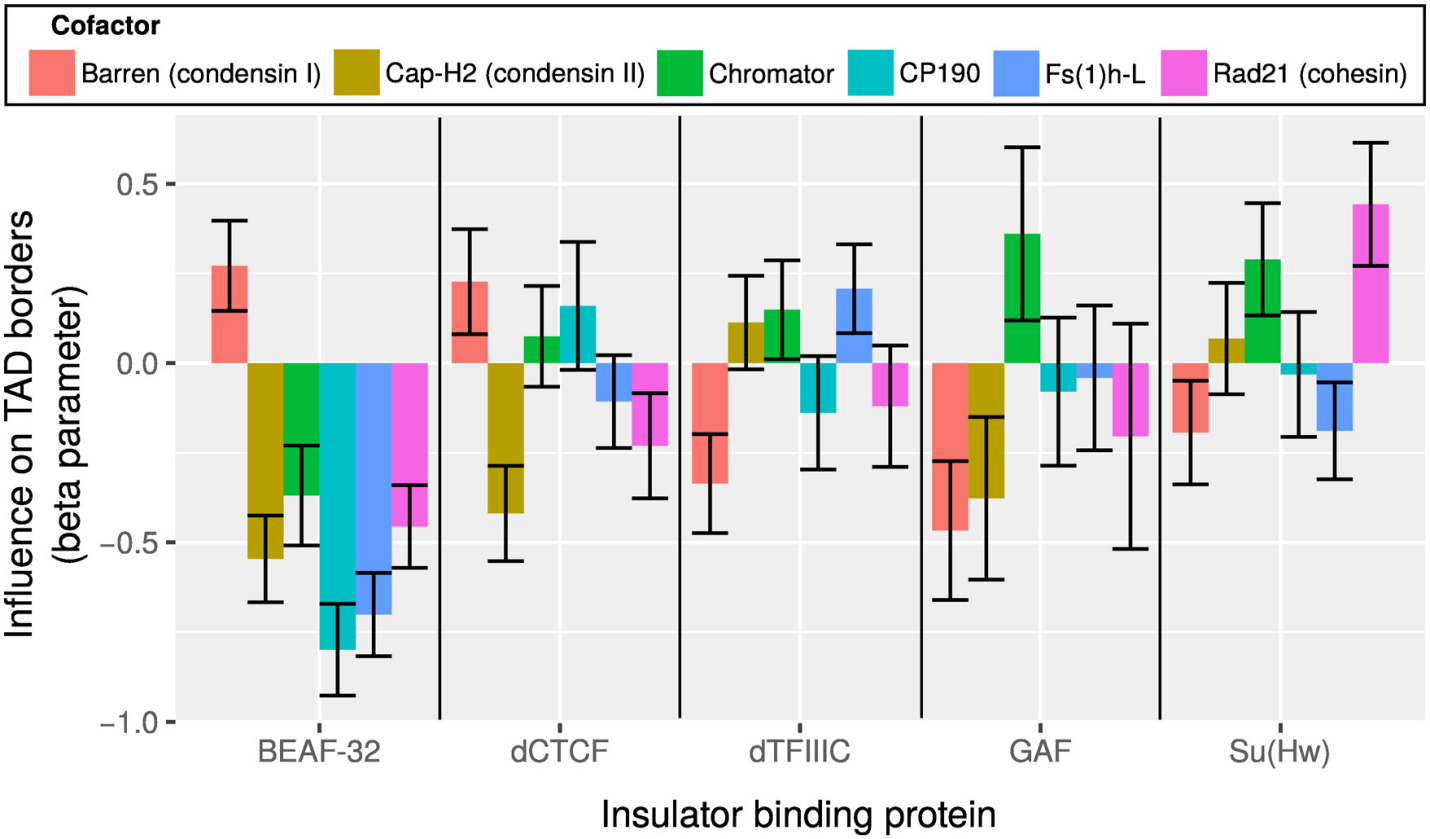
Analysis of interactions between insulator binding proteins (IBPs) and cofactors at topologically associating domain (TAD) borders of wild-type *Drosophila* Kc167 cells. Beta parameter corresponding to each interaction IBP-cofactor from the multiple logistic regression is plotted. Interaction terms are detailed in Subsection Materials and Methods, Analysis of interactions. Error bars show 95% confidence intervals of beta parameters. Barren is a subunit of condensin I, Cap-H2 is a subunit of condensin II and Rad21 is a subunit of cohesin.

An important question is to know if IBPs demarcate TAD borders depending on the presence of specific cofactors [10]. To answer this question, we assessed if the co-occurence of an IBP with a cofactor could affect TAD borders by estimating the corresponding statistical interaction IBP-cofactor (Fig 6). Among the significant positive interactions, we reported effects for Su(Hw) with Rad21 ($\hat{\beta }=0.44$, *p* = 3 × 10^−7^), and lower effects of Su(Hw) with Chromator ($\hat{\beta }=0.29$, *p* = 2 × 10^−4^), BEAF-32 with condensin I (Barren) ($\hat{\beta }=0.27$, *p* = 2 × 10^−5^), dTFIIIC with Fs(1)h-L ($\hat{\beta }=0.21$, *p* = 0.001), dCTCF with condensin I (Barren) ($\hat{\beta }=0.23$, *p* = 2 × 10^−3^). These positive interactions reflected synergistic effects of IBPs with cofactors. We did not report any significant positive statistical interaction between dCTCF and cohesin as observed in human [8]. In contrast to vertebrates, *Drosophila* CTCF does not appear to rely on cohesin to establish or maintain interactions [42]. Of interest, our method further highlighted strong and significant negative interactions that revealed antagonistic effects at domain borders, in particular for BEAF-32 with cofactor CP190 ($\hat{\beta }=-0.80$, *p* < 1 × 10^−20^). As such, our model may allow to retrieve both synergistic and antagonistic influences of co-factors, which may better reflect the complexity behind the establishment or maintenance of TAD borders.

### Analysis of functional elements in *Drosophila*

**Fig 7:**
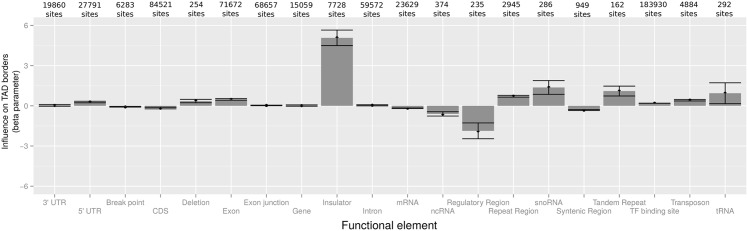
Analysis of functional elements using multiple logistic regression at topologically associating domain (TAD) borders of wild-type *Drosophila* Kc167 cells. Error bars show 95% confidence intervals of beta parameters.

We sought to further investigate a wide variety of functional elements such as insulators and regulatory sequences. Results are reported in Fig 7. Insulators were by far the most influential functional elements with respect to domain borders ($\hat{\beta }=5.07$, *p* < 1 × 10^−20^), as established in human [8, 31]. Regarding other functional elements, we found positive effects for repeat regions ($\hat{\beta }=0.71$, *p* < 1 × 10^−20^), and especially for tandem repeats on TAD borders ($\hat{\beta }=1.10$, *p* = 5 × 10^−9^). Repeat regions were previously reported to spatially cluster together [43]. In addition, snoRNA genes had a positive influence on domain borders ($\hat{\beta }=1.37$, *p* = 1 × 10^−7^), which may reflect their role in higher-order chromatin structure [44]. Furthermore, a negative impact on TAD border was detected for regulatory sequences ($\hat{\beta }=1.87$, *p* = 6 × 10^−10^), strengthening the hypothesis that functional long-range contacts involving regulatory elements could compete with structural contacts [45] (see Discussion).

### Positive and negative effects of proteins in human

We next analyzed the effects of DNA-binding proteins on 3D domains of human genome where fewer architectural proteins have been uncovered [29]. To investigate the possible contributions of these proteins, we analyzed new 3D domains detected from recent high resolution Hi-C data at 1 kb for GM12878 cells for which a large number of ChIP-seq data were available [8]. Over the 69 proteins analyzed, 51 proteins presented very high and significant enrichments (all coefficients $\hat{\beta }>3$ and all p-values *p* < 1 × 10^−20^). Multiple logistic regression instead detected 15 proteins with significant positive effects on domain borders (all coefficients $\hat{\beta }>0.5$ and all p-values *p* < 5 × 10^−4^; S3 Table). Our analyses confirmed that, in contrast to *Drosophila*, CTCF and cohesin (subunit Rad21) presented the highest effects among all factors (CTCF: $\hat{\beta }=1.90$, *p* < 1 × 10^−20^; cohesin: $\hat{\beta }=1.91$, *p* < 1 × 10^−20^), in complete agreement with numerous studies showing their important roles in shaping chromosome 3D structure in mammals [8, 9, 12]. ZNF143 had the third highest effect ($\hat{\beta }=1.85$, *p* < 1 × 10^−20^), in total agreement with a very recent study demonstrating its role in long-range contacts [46]. In addition, multiple logistic regression identified EZH2, the catalytic subunit of the Polycomb repressive complex 2 (PRC2), as a protein that significantly impacted TAD borders (4th highest effect: $\hat{\beta }=1.32$, *p* < 5 × 10^−11^). In contrast, multiple logistic regression estimated a null beta for candidate architectural proteins JUND ($\hat{\beta }=0.04$, *p* = 0.85), Kaiso ($\hat{\beta }=0.43$, *p* = 0.10) and a very low beta for MAZ ($\hat{\beta }=0.23$, *p* = 3 × 10^−4^). Although these three proteins colocalize or interact with CTCF, our model suggests that they might not impact TAD borders. We also notably identified several factors associated with transcriptional activation that had significant negative influences on TAD borders. These proteins included RXRA ($\hat{\beta }=-1.37$, *p* = 3 × 10^−4^), P300 ($\hat{\beta }=-1.22$, *p* = 1 × 10^−10^), BCL11A ($\hat{\beta }=-0.82$, *p* = 1 × 10^−9^) and ELK1 ($\hat{\beta }=-0.74$, *p* = 4 × 10^−9^), reinforcing the view that transcription could also interfere with TAD borders depending on context.

### Large-scale analysis of DNA motifs in human

In the previous subsection, analyses of DNA-binding proteins were limited by available ChIP-seq data. Here we alleviated this limitation by analyzing transcription factor binding site (TFBS) motifs available from the large MotifMap database [47]. Given the large number of TFBS motifs (544 motifs), we used L1-regularization for parameter estimation. We identified 213 positive drivers (all coefficients $\hat{\beta }>1$) and 75 negative drivers (all coefficients $\hat{\beta }<1$), meaning that a large number of TFBSs actually play a role in TAD border establishment or maintenance. CTCF motifs ranked first ($\hat{\beta }=45.34$) in complete agreement with recent studies [8, 31]. But our model also uncovered other TFBSs whose roles in TAD borders are less well known such as EGR-1 ($\hat{\beta }=34.04$), p53 ($\hat{\beta }=25.55$), MIZF ($\hat{\beta }=22.46$), GABP ($\hat{\beta }=21.94$) and many others (for a complete list, see S4 Table). For instance, p53 is a major tumor suppressor gene and the most frequently mutated gene (>50%) in human cancer [48]. Regarding negative drivers, we identified ALX4 ($\hat{\beta }=-35.82$), EGR4 ($\hat{\beta }=-26.72$), ZNF423 ($\hat{\beta }=-23.97$). All these results highlighted the great potential of TFBS motif analysis allowing the study of a very large number of DNA-binding proteins.

## Discussion

Here, we describe a multiple logistic regression (MLR) to assess the roles of genomic features such as DNA-binding proteins and functional elements on TAD border establishment/maintenance. Based on conditional independence, such regression model can identify genomic features that impact TAD borders, unlike enrichment test (ET) and non-parametric models. Using simulations, we demonstrate that model parameters can be accurately estimated for both marginal genomic features (no interaction) and two-way interactions. In addition, we show that our model outperforms enrichment test and random forests for the identification of genomic features that influence domain borders. Using recent experimental Hi-C and ChIP-seq data, the proposed model can identify genomic features that are most influential with respect to TAD borders at a very high resolution of 1 kb in both *Drosophila* and human. The proposed model could thus guide the biologists for the design of most critical Hi-C experiments aiming at unraveling the key molecular determinants of higher-order chromatin organization.

**Fig 8:**
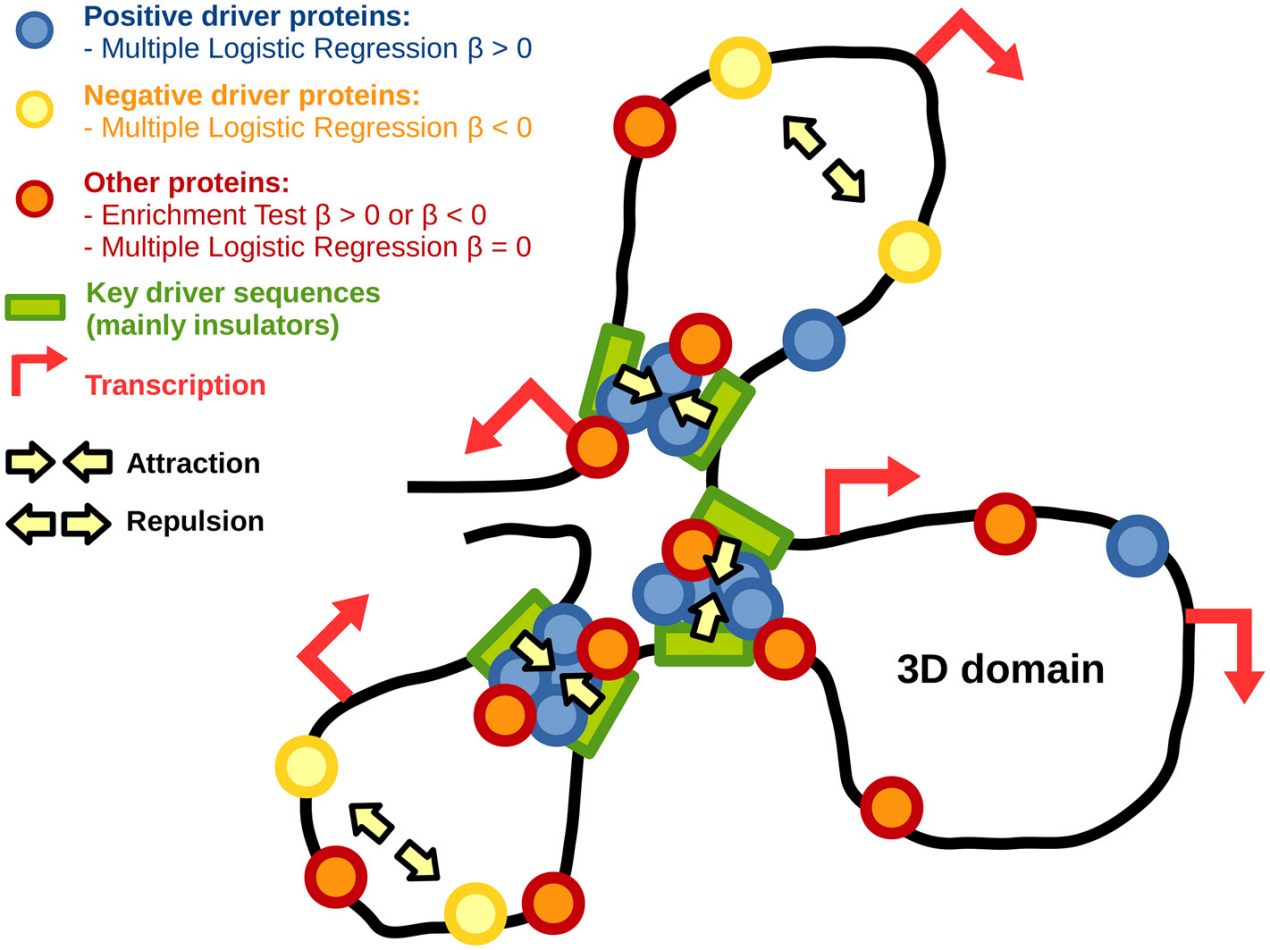
Model for 3D domain border establishment or maintenance.

Enrichment test shows slight differences of enrichments among architectural proteins. This could suggest that domain borders are determined by the number and levels of all proteins present at the border rather than the presence of specific proteins [11, 13]. However MLR instead reveals that only some architectural proteins influence the presence of 3D domain borders. Moreover, MLR retrieves both positive and negative contributions among most influencial proteins, depending on contexts such as co-occurence. From these novel results, we propose a biological model for 3D domain border establishment or maintenance (Fig 8). In this model, three kinds of proteins are distinguished: positive drivers (*β*_*MLR*_ > 0), negative drivers (*β*_*MLR*_ < 0), and proteins that are enriched or depleted at borders but are not drivers (*β*_*ET*_ > 0 or *β*_*ET*_ < 0, and *β*_*MLR*_ = 0). Positive drivers favor attraction between domain borders leading to the formation of 3D domains. CTCF and cohesin are well-studied positive drivers in mammals [8, 10]. By contrast little is known about negative drivers of 3D domain borders that could favor repulsion between specific chromatin regions [49]. Repulsion phenomenon could be the result of allosteric effects of loops in chromatin [45]. Negative drivers could also regulate disassembly of protein complex that mediate long-range contacts [50].

In *Drosophila*, MLR identifies BEAF-32, a well-characterized IBP, as a positive driver of TAD borders [51, 52]. Conversely, other IBPs including dCTCF, dTFIIIC, GAF and Su(Hw) are found significantly enriched at TAD borders, but present weak or no influences, in agreement with recent works [53]. Regarding cofactors, CP190 presents a high and significant positive influence on domain demarcation, in agreement with previous findings [5]. Regarding functional elements, although our data highlight that insulators are by far the main positive drivers of TAD borders, they also show that additional elements, that are known to colocalize in 3D [18, 43, 44], play a role including repeat regions. Moreover, MLR suggests that snoRNA genes are novel functional elements that positively influence border demarcation. Recent works suggest that active chromatin and transcription also play a key role in chromosome partitioning in TADs [53]. Here our results reveal that both architectural proteins and transcription contribute to TAD borders. In contrast, regulatory regions are identified as negative drivers of TAD borders. One possible explanation is that such regulatory regions are involved in functional long-range contacts with gene promoters that would compete with the formation of more structural contacts at the origin of TADs [45]. Alternatively, a negative influence may be linked to the transient nature of certain functional contacts [54].

Almost half of dCTCF and cohesin sites are overlapping in *Drosophila*, and knockdown of dCTCF results in a strong decrease of cohesin binding [11]. As such, one might expect synergistic effects of dCTCF with cohesin (also called statistical interaction) in driving TAD borders. However, such conclusion could not be drawn. Following statistical theory, it is not because two variables are correlated (here dCTCF and cohesin colocalize), that it implies a synergistic effect of the two variables on TAD borders. Although dCTCF and cohesin are both enriched at TAD borders, MLR does not detect a significant interaction of dCTCF with cohesin. Instead we observe a high interaction of Su(Hw) with cohesin. Negative interactions that reflect antagonistic effects between architectural proteins are found between IBP BEAF-32 and cofactor CP190. These antagonistic effects suggest that cofactors might not always help IBPs in stabilizing loops [10]. One explanation is that cofactors could sometimes compete with IBPs for long-range protein-protein interactions.

In human, MLR identifies well-studied architectural proteins CTCF and cohesin as the most influential positive drivers of 3D domains, in complete agreement with their established roles in shaping chromosome 3D structure [8, 9, 12]. MLR also points out the positive influences of ZNF143 and PRC2 proteins whose recent studies have uncovered their roles in controlling spatial organization [30, 46]. In addition, our model reveals the roles of additional factors including RXRA, P300, BCL11A and ELK1 as negative drivers of 3D domain borders. P300 was previously shown to be depleted at domain borders [55]. Here we find that P300 and three other proteins can counteract the establishment or maintenance of domain borders. P300 is a well-known regulator of cell growth and division, and helps prevent the growth of cancerous tumors [56]. Interestingly, the three other proteins RXRA, BCL11A and ELK1 are also related to cancer [57–59]. Furthermore, the analysis of a large number of TFBS motifs confirmed the role of CTCF in TAD border formation [8, 31]. But this analysis also uncovered many other TFBSs, such as p53, a major tumor suppressor gene [48].

The proposed method relies on the accurate identification of 3D domains. To further improve our understanding of the key drivers of 3D domain borders, Hi-C experiments at a higher resolution are needed. In addition, a variety of methods have been recently developed for 3D domain inference, and no consensus has been reached yet to determine which method is the most appropriate. Another important question is to understand the roles of key drivers in chromatin interactions within domains. For instance, it is essential to identify proteins that influence functional interactions between enhancers and promoters that regulate gene expression. Although far more complex, it is of note that similar regression approach may largely help in retrieving positive from negative patterns in these contexts.

## Materials and Methods

### Hi-C data and topologically associating domains

For *Drosophila* 3D domain analysis, we used publicly available high-throughput chromatin conformation capture (Hi-C) data from Gene Expression Omnibus (GEO) accession GSE63515 [13]. Hi-C experiments were done for wild-type *Drosophila melanogaster* Kc167 cells with DpnII restriction enzyme. Hi-C data were binned at 1 kb resolution. Contact matrices were normalized using ICE method [15] implemented in the R package HiTC (http://www.bioconductor.org/packages//2.11/bioc/html/HiTC.html). From the normalized contact matrices, TAD genomic coordinates were identified using HiCseg method [19].

For human 3D domain analysis, we used publicly available 3D domains of GM12878 cells identified by the Arrowhead algorithm from Gene Expression Omnibus (GEO) accession GSE63525 [8].

### ChIP-seq data

For *Drosophila* analysis, we used publicly available binding profiles of chromatin proteins of *Drosophila melanogaster* wild-type embryonic Kc167 cells. ChIP-seq data for CP190, Su(Hw), dCTCF and BEAF-32 were obtained from GEO accession GSE30740 [60]. ChIP-seq data for Barren (condensin I), Cap-H2 (condensin II), Chromator, Rad21 (cohesin), GAF and dTFIIIC were obtained from GEO accession GSE54529 [11]. ChIP-seq data for Fs(1)h-L and Fs(1)h-LS were obtained from GEO accession GSE42086 [38]. ChIP-seq peaks were called using MACS 1.4.2 (https://github.com/taoliu/MACS). Fs(1)h-S peaks were defined as peaks from Fs(1)h-LS that did not overlap any Fs(1)h-L peak.

For human analysis, we used publicly available ChIP-seq peaks of 69 chromatin proteins (ATF2, ATF3, BATF, BCL11A, BCL3, BCLAF1, BHLHE40, BRCA1, CEBPB, CHD1, CHD2, CTCF, E2F4, EBF1, EGR1, ELF1, ELK1, ETS1, EZH2, FOS, FOXM1, IKZF1, IRF3, IRF4, JUND, MAFK, MAX, MAZ, MEF2A, MEF2C, MTA3, MXI1, MYC, NFATC1, NFE2, NFIC, NFYA, NFYB, NRF1, P300, PAX5, PBX3, PIGG, PML, POU2F2, RAD21, REST, RFX5, RUNX3, RXRA, SIN3A, SIX5, SP1, SRF, STAT1, STAT3, STAT5A, TAF1, TCF12, TCF3, USF1, USF2, YY1, ZBTB33, ZEB1, ZNF143, ZNF274, ZNF384 and ZZZ3) of GM12878 cells from ENCODE [61].

### Functional elements

For *Drosophila* analysis, we used RNA-seq data from wild-type Kc167 cells to map active transcription start sites (TSSs) [62]. For all other functional elements, we used flybase reference genome annotation (http://flybase.org/).

### DNA motifs

For human analysis, we used transcription factor binding site (TFBS) motifs from the MotifMap database (http://motifmap.ics.uci.edu/).

### Binned data matrix

From TAD coordinates, ChIP-seq data and functional element mapping, we constructed 50-base and 1-kb binned data matrices that were further used for multiple logistic regressions with *Drosophila* and human data, respectively. A matrix was composed of a column variable *Y* that indicated if the genomic bin belonged to a TAD boundary (*Y* = 1) or not (*Y* = 0). To define TAD boundaries, we extracted 1 kb and 20 kb regions that were centered around the positions demarcating two TADs in *Drosophila* and human genomes, respectively. The other column variables **X** = {*X*_1_, …, *X*_*p*_} were the set of *p* genomic feature variables of interest. If genomic coordinate data were used (*e.g.*, ChIP-seq peak or functional element coordinates), variable *X*_*i*_ denoted the presence (*X*_*i*_ = 1) or absence (*X*_*i*_ = 0) of the genomic feature *i* within the genomic bin. Note that if a genomic coordinate only overlapped *x*% of the genomic bin, then *X*_*i*_ = *x*%. If quantitative data were used (*e.g.*, ChIP-seq signal intensity log(*ChIP/Input*)), variable *X*_*i*_ was the average value within the genomic bin.

### Enrichment test

Enrichment test assesses the enrichment of a genomic feature within chromatin domain borders. The genomic feature of interest can be protein-DNA binding sites detected from ChIP-seq experiment. Chromatin domain borders can be borders between topologically associating domains identified from Hi-C experiment.

From the contingency table (Table 1), one can test the odds ratio that reflects the magnitude of enrichment (*OR* > 1) or depletion (*OR* < 1) of the genomic feature within the domain borders. The test consists in assessing the following null (*H*_0_) and alternative (*H*_1_) hypotheses about odds ratio *OR*:

$$H_{0}:OR=1$$ (2)

$$H_{1}:OR\neq 1$$ (3)

The odds ratio is the ratio of the inside border odds (500/5000) to the outside border odds (2000/2000000). Here $\hat{OR}=\frac{500/5000}{2000/200000}=10$.

**Table 1:** Example of a contingency table to assess enrichment (or depletion) of a genomic feature within the domain borders.

	Presence of the feature	Absence of the feature
Inside border	500	5000
Outside border	2000	200000

Previous enrichment test can be reformulated as a simple logistic regression model:

$$\text{ln}\frac{Prob(Y=1|X_{i})}{1-Prob(Y=1|X_{i})}=\beta_{0}+\beta X_{i}$$ (4)

Variables *X*_*i*_ ∈ **X** and *Y* are described in Subsection Materials and Methods, Binned data matrix. In the simple logistic regression, the slope parameter *β* is the natural logarithm of the abovementioned odds ratio *OR*. Thus *β* > 0 means enrichment, while *β* < 0 reflects depletion. Using logistic regression model, parameter *β* can be tested by Wald’s test. The Wald’s statistic is calculated as:

$$W=\frac{\hat{\beta }-\beta_{*}}{{\hat{\sigma }}_{\beta }}=\frac{\hat{\beta }-0}{{\hat{\sigma }}_{\beta }}=\frac{\hat{\beta }}{{\hat{\sigma }}_{\beta }}$$ (5)

Where β_*_ is the beta parameter value under *H*_0_ assumption (β_*_ = 0) and ${\hat{\sigma }}_{\beta }$ denotes the standard error of parameter *β*. Statistic *W* follows a normal distribution.

An important drawback of enrichment test relies on the fact that it does not account for potential colocalizations (*i.e.* correlations) among the genomic features of interest. The presence of correlations might prevent the identification of the genomic features that really drive the establishment or maintenance of domain borders. For instance, if two genomic features are significantly enriched, this might not mean that both are involved in the establishment or maintenance of the borders. One feature might truly affect borders while the other feature might only be correlated to the former. There is thus a need for a model that could identify those enriched features that drive the presence of borders.

### Multiple logistic regression

The proposed multiple logistic regression is an extension of the simple logistic regression for *p* genomic features:

$$\text{ln}\frac{Prob(Y=1|X)}{1-Prob(Y=1|X)}=\beta_{0}+\beta X$$ (6)

Where **X** = {*X*_1_, …, *X*_*p*_} is the set of *p* genomic features of interest and ***β*** = {*β*_1_, …, *β*_*p*_} denotes the set of slope parameters (one parameter for each genomic feature). As for simple logistic regression, each *β*_*i*_ ∈ ***β*** coefficient can be tested by a Wald’s test.

By default, multiple logistic regression *β*_0_ and ***β*** parameters are estimated by iteratively reweighted least squares. However, when there are a large number of correlated genomic features in the model, L1-regularization is applied and parameters are learned by coordinate descent [26]. The L1-regularization lambda that gives the lowest mean cross-validated error is selected. To assess quality of fit for a model, we use the deviance ratio defined as the ratio of the fitted model deviance to the saturated model deviance. We also use Akaike information criterion (AIC).

The matrix **X** is sparse and the Wald’s test might be biased when data are sparse [27]. Hence likelihood ratio test (LRT) that is not affected by data sparseness can be used instead. To test parameter *β*_*i*_ with LRT, two models are built: a first model $M_{1}$ over all variables **X**, and a second model $M_{2}$ over all variables except *X*_*i*_ (**X** \ *X*_*i*_). Then the following *D*_*i*_ statistic is calculated:

$$D_{i}=-2\text{ln}\left(\frac{L_{M_{1}}}{L_{M_{2}}}\right)$$ (7)

Where $L_{M_{1}}$ is the likelihood of $M_{1}$ and $L_{M_{2}}$ is the likelihood of $M_{2}$. Statistic *D*_*i*_ follows a chi-squared distribution with one degree of freedom. The better accuracy of LRT comes at the cost of more intensive computations. In practice, we observe that Wald’s test p-values are close to LRT p-values.

In the multiple logistic regression setting, parameter *β*_*i*_ measures the effect of genomic feature *X*_*i*_ on the presence of borders conditional on the other genomic features that belong to **X** \ *X*_*i*_. A value of *β*_*i*_ > 0 or *β*_*i*_ < 0 means that the genomic feature *X*_*i*_ positively or negatively influences the presence of borders, respectively. A value of *β*_*i*_ = 0 reflects the fact that the genomic feature *X*_*i*_ does not affect the presence of borders. If two genomic features *X*_1_ and *X*_2_ are colocalized and only *X*_1_ drives the establishment or maintenance of domain borders, then only the corresponding *β*_1_ parameter will be significantly different from zero. However the above formulation of the model does not account for potential statistical interactions between genomic features.

### Analysis of interactions

Interaction terms can be included in the multiple logistic regression to account for potential interactions between genomic features. For instance, one can include in the model an interaction term between two genomic features *X*_1_ and *X*_2_:

$$\text{ln}\frac{Prob(Y=1|X_{1},X_{2})}{1-Prob(Y=1|X_{1},X_{2})}=\beta_{0}+\beta_{1}X_{1}+\beta_{2}X_{2}+\beta_{12}X_{1}X_{2}$$ (8)

The product *X*_1_ *X*_2_ is the statistical interaction term between the two genomic features *X*_1_ and *X*_2_. Parameter *β*_12_ measures the effect of interaction *X*_1_ *X*_2_ on the presence of borders.

### Data simulation

In order to assess the accuracy of multiple logistic regression parameter estimation, we simulated data that were the most similar to the real genomic data using the following procedure. First, for a simulation *s*, a set of observation rows was randomly drawn with resampling from matrix **X** (nonparametric bootstrap). This resampling allowed to keep the original correlation structure among the variables. The bootstrapped data matrix was denoted **X**^*s*^. Second $\beta^{s}=\left\{\beta_{1}^{s},...,\beta_{p}^{s}\right\}$ parameter values were drawn from a normal distribution N(μ,σ) with mean *μ* = 0 and variance *σ* = 1. Parameter $\beta_{0}^{s}$ (intercept) value was drawn from a normal distribution with same variance but with mean *μ* = −4.5. This setting of the mean of $\beta_{0}^{s}$ allowed to control the number of values *Y* = 1 close to the one observed from real data (the number of borders in real data was low). Third a quantitative variable *Z*^*s*^ was calculated using the regression formula: $Z^{s}=\beta_{0}^{s}+\beta^{s}X^{s}$. A probability variable *Prob*^*s*^ was calculated by the inverse logit function: 1/(1 + *exp*(−*Z*^*s*^)). Then each probability value from *Prob*^*s*^ was used to draw a value for *Y*^*s*^ using binomial distribution.

We also used simulated data to compare multiple logistic regression with enrichment test and random forests. As previously, for a simulation *s*, we used non-parametric bootstrap and kept the correlation structure of original data. Among the variables, a subset of variables **X**_*c*_ ∈ **X** was chosen to be causal, *i.e.* to influence the presence of borders. We chose a generative model that was non-linear and non-additive not to favor multiple logistic regression over other models. For this purpose, we set a probability *p*_0_ of the presence of a border in a bin if all causal variable values were inferior to 0.5. We also set a probability *p*_1_ (with *p*_1_ > *p*_0_) if at least one causal variable had a value superior or equal to 0.5. Values of *p*_0_ and *p*_1_ were chosen according to the number of borders in real data. Then, for each bin, the value for *Y*^*s*^ was drawn using a binomial distribution with either *p*_0_ or *p*_1_ depending on the causal variable values.

### Implementation and availability

The multiple logistic regression is implemented in R language. The model is available in the R package “HiCfeat” which can be downloaded from the Comprehensive R Archive Network and from the web page of Raphaël Mourad (https://sites.google.com/site/raphaelmouradeng/home/programs).

## Supporting Information

S1 Table Deviance ratios and Akaike information criteria obtained for multiple logistic regression models in wild-type *Drosophila* Kc167 cells. (PDF)

S2 Table Multiple logistic regression including insulator-binding proteins (IBPs), cofactors and IBP-cofactor interactions at topologically associating domain borders of wild-type *Drosophila* Kc167 cells. (PDF)

S3 Table Multiple logistic regression including DNA-binding proteins in human GM12878 cells at 3D domain borders. Here 3D domains identified by the Arrowhead algorithm were used.
(PDF)

S4 Table Multiple logistic regression including transcription factor binding site (TFBS) motifs in human GM12878 cells at 3D domain borders. Here 3D domains identified by the Arrowhead algorithm were used.
(PDF)

S1 Fig Parameter estimation accuracy of multiple logistic regression for simulated proteins with varied numbers of ChIP-seq peaks. (PDF)

S2 Fig Impact of the inaccuracy of topologically associating domain (TAD) borders on multiple logistic regression beta parameters. R squared is computed between beta parameters estimated from TAD borders and beta parameters estimated from TAD borders with random noise. Random noise was drawn from a normal distribution of mean zero and varying standard deviations in kb (x-axis).
(PDF)

S3 Fig Comparison of multiple logistic regression (MLR) with enrichment test (ET) and random forests (RF) to detect known and suspected architectural proteins in human using GM12878 cell ChIP-seq data binned at 40 kb resolution. Receiver operating characteristic (ROC) curves were computed from Wald’s statistics for ET, beta parameters for MLR, and variable importances for random forests.
(PDF)

S4 Fig Comparison of MLR with ET and RF to detect the influences of single nucleotide polymorphisms (SNPs) in the CTCF motif on 3D domains in human. Receiver operating characteristic (ROC) curves were computed from Wald’s statistics for ET, from beta parameters for MLR, and from variable importances for random forests. Computations were carried out at 1 kb resolution.
(PDF)

S5 Fig Analysis of the impacts of single nucleotide polymorphisms on the consensus BEAF-32 motif in wild-type *Drosophila* Kc167 cells. (PDF)

S6 Fig Comparison of the influences of transcription and of architectural proteins on topologically associating domain borders of wild-type *Drosophila* Kc167 cells. a) Multiple logistic regression of active TSSs and BEAF-32. b) Multiple logistic regression of Fs(1)h-S and Fs(1)h-L.
(PDF)

S7 Fig Multiple logistic regression of cofactors at topologically associating domain borders of wild-type *Drosophila* Kc167 cells. (PDF)
