# Supplementary material for: Computational Identification of Genomic Features That Influence 3D Chromatin Domain Formation
Source: PLoS Comput Biol. 2016 May 20;12(5):e1004908. doi: 10.1371/journal.pcbi.1004908 (PMC4874696; doi:10.1371/journal.pcbi.1004908)
Supplement: S1 Table — (PDF) [file pcbi.1004908.s001.pdf]

Model	Statistical Interactions	Deviance Ratio		Akaike Information Criterion	
		Genomic Coordinate Data	Quantitative Data	Genomic Coordinate Data	Quantitative Data
Insulator binding proteins	No	0.100	0.068	152806	158402
Cofactors	No	0.120	0.073	149399	157517
Insulator binding proteins + Cofactors	No	0.126	0.089	148499	154722
Insulator binding proteins + Cofactors	Yes	0.135	0.108	146918	151605
Functional elements	No	0.073	NA	157521	NA
