# Supplementary material for: Computational Identification of Genomic Features That Influence 3D Chromatin Domain Formation
Source: PLoS Comput Biol. 2016 May 20;12(5):e1004908. doi: 10.1371/journal.pcbi.1004908 (PMC4874696; doi:10.1371/journal.pcbi.1004908)
Supplement: S2 Table — (PDF) [file pcbi.1004908.s002.pdf]

Protein	Beta	Standard Error	z value	P-value
BEAF-32	2.01	0.05	42.83	$< 1 \times 10^{-20}$
dCTCF	0.24	0.09	2.69	0.009
dTFIIIC	0.41	0.10	4.28	3×10^{-5}
GAF	0.86	0.15	5.64	3×10^{-7}
Su(Hw)	-0.69	0.09	-7.59	4×10^{-16}
Barren	-0.04	0.05	-0.79	0.42
Cap-H2	1.14	0.05	21.22	$< 1 \times 10^{-20}$
Chromator	0.32	0.07	4.63	4×10^{-6}
CP190	1.13	0.05	22.25	$< 1 \times 10^{-20}$
Fs(1)h-L	0.86	0.05	18.45	$< 1 \times 10^{-20}$
Rad21	0.83	0.03	26.11	$< 1 \times 10^{-20}$
BEAF-32 x Barren	0.27	0.06	4.23	2×10^{-5}
dCTCF x Barren	0.23	0.07	3.04	0.002
dTFIIIC x Barren	-0.34	0.07	-4.77	2×10^{-6}
GAF x Barren	-0.47	0.10	-4.73	2×10^{-6}
Su(Hw) x Barren	-0.19	0.07	-2.63	0.008
BEAF-32 x Cap-H2	-0.55	0.06	-8.85	$< 1 \times 10^{-20}$
dCTCF x Cap-H2	-0.42	0.07	-6.19	6×10^{-10}
dTFIIIC x Cap-H2	0.11	0.07	1.70	0.09
GAF x Cap-H2	-0.38	0.12	-3.26	0.001
Su(Hw) x Cap-H2	0.07	0.08	0.86	0.38
BEAF-32 x Chromator	-0.37	0.07	-5.20	2×10^{-7}
dCTCF x Chromator	0.07	0.07	1.04	0.3
dTFIIIC x Chromator	0.15	0.07	2.12	0.03
GAF x Chromator	0.36	0.12	2.93	0.004
Su(Hw) x Chromator	0.29	0.08	3.62	3×10^{-4}
BEAF-32 x CP190	-0.80	0.07	-12.25	$< 1 \times 10^{-20}$
dCTCF x CP190	0.16	0.09	1.75	0.08
dTFIIIC x CP190	-0.14	0.08	-1.72	0.08
GAF x CP190	-0.08	0.11	-0.76	0.45
Su(Hw) x CP190	-0.03	0.09	-0.36	0.72
BEAF-32 x Fs(1)h-L	-0.70	0.06	-11.83	$< 1 \times 10^{-20}$
dCTCF x Fs(1)h-L	-0.11	0.07	-1.63	0.10
dTFIIIC x Fs(1)h-L	0.21	0.06	3.28	9×10^{-4}
GAF x Fs(1)h-L	-0.04	0.10	-0.40	0.69
Su(Hw) x Fs(1)h-L	-0.19	0.07	-2.74	0.006
BEAF-32 x Rad21	-0.46	0.06	-7.75	1×10^{-14}
dCTCF x Rad21	-0.23	0.07	-3.08	0.002
dTFIIIC x Rad21	-0.12	0.09	-1.39	0.17
GAF x Rad21	-0.20	0.16	-1.27	0.21
Su(Hw) x Rad21	0.44	0.09	5.06	3×10^{-7}
