# Supplementary material for: Computational Identification of Genomic Features That Influence 3D Chromatin Domain Formation
Source: PLoS Comput Biol. 2016 May 20;12(5):e1004908. doi: 10.1371/journal.pcbi.1004908 (PMC4874696; doi:10.1371/journal.pcbi.1004908)
Supplement: S3 Table — Here 3D domains identified by the Arrowhead algorithm were used. (PDF) [file pcbi.1004908.s003.pdf]

Protein	Beta	Standard Error	z value	P-value
RAD21	1.905	0.132	14.382	$< 1 \times 10^{-20}$
CTCF	1.900	0.061	30.996	$< 1 \times 10^{-20}$
ZNF143	1.853	0.087	21.293	< 1 x 10-19
EZH2	1.319	0.200	6.596	4×10^{-11}
BRCA1	1.129	0.441	2.563	0.010
YY1	0.983	0.065	15.071	$< 1 \times 10^{-20}$
ZZZ3	0.910	0.329	2.767	0.006
ZNF384	0.905	0.041	22.190	$< 1 \times 10^{-20}$
RUNX3	0.790	0.052	15.227	< 1 x 10-19
BATF	0.708	0.111	6.359	2×10^{-10}
EBF1	0.698	0.080	8.760	2×10^{-18}
SIN3A	0.612	0.063	9.670	$< 1 \times 10^{-20}$
NFE2	0.604	0.421	1.435	0.151
MXI1	0.582	0.072	8.113	5×10^{-16}
EGR1	0.574	0.122	4.717	2×10^{-6}
NFYB	0.571	0.085	6.692	2×10^{-11}
USF1	0.533	0.155	3.448	6×10^{-4}
ELF1	0.518	0.077	6.758	2×10^{-11}
NRF1	0.510	0.164	3.117	0.002
CHD1	0.504	0.086	5.885	2×10^{-9}
Kaiso	0.433	0.262	1.655	0.098
NFYA	0.410	0.272	1.504	0.133
PAX5	0.402	0.096	4.203	3×10^{-5}
SRF	0.371	0.164	2.256	0.024
PBX3	0.353	0.155	2.282	0.022
TAF1	0.264	0.102	2.594	0.009
MAZ	0.234	0.065	3.604	3×10^{-4}
MAFK	0.234	0.097	2.400	0.016
STAT1	0.234	0.157	1.490	0.136
ZEB1	0.224	0.136	1.651	0.099
FOXM1	0.221	0.056	3.956	8×10^{-5}
BCL3	0.219	0.094	2.317	0.020
USF2	0.201	0.147	1.370	0.171
POU2F2	0.172	0.079	2.174	0.030
ATF3	0.150	0.418	0.360	0.719
NFATC1	0.145	0.063	2.319	0.020
FOS	0.076	0.322	0.236	0.814
MYC	0.054	0.081	0.668	0.504
MTA3	0.049	0.059	0.841	0.400
JUND	0.042	0.225	0.186	0.853
ATF2	0.025	0.062	0.408	0.683
PIGG	0.022	0.084	0.258	0.796
STAT5A	0.019	0.073	0.259	0.796
NFIC	0.002	0.051	0.043	0.966
TCF12	-0.036	0.115	-0.313	0.754
PML	-0.050	0.051	-0.963	0.336
IKZF1	-0.122	0.066	-1.859	0.063
STAT3	-0.139	0.107	-1.307	0.191
IRF4	-0.141	0.099	-1.418	0.156
TCF3	-0.172	0.108	-1.597	0.110
MAX	-0.201	0.081	-2.466	0.014
CEBPB	-0.202	0.100	-2.019	0.044
IRF3	-0.222	0.113	-1.956	0.051
E2F4	-0.247	0.161	-1.532	0.126
MEF2C	-0.254	0.146	-1.737	0.082
MEF2A	-0.261	0.120	-2.180	0.029
RFX5	-0.309	0.151	-2.049	0.041
BCLAF1	-0.318	0.102	-3.115	0.002
SP1	-0.337	0.104	-3.244	0.001
CHD2	-0.347	0.090	-3.847	1×10^{-4}
BHLHE40	-0.407	0.101	-4.021	6×10^{-5}
SIX5	-0.429	0.207	-2.069	0.039
ETS1	-0.561	0.231	-2.432	0.015
REST	-0.596	0.179	-3.331	9×10^{-4}
ELK1	-0.739	0.126	-5.885	4×10^{-9}
BCL11A	-0.816	0.134	-6.088	1×10^{-9}
P300	-1.220	0.192	-6.345	2×10^{-10}
RXRA	-1.365	0.381	-3.579	3×10^{-4}
ZNF274	-2.592	1.066	-2.432	0.015
