# Supplementary material for: Computational Identification of Genomic Features That Influence 3D Chromatin Domain Formation
Source: PLoS Comput Biol. 2016 May 20;12(5):e1004908. doi: 10.1371/journal.pcbi.1004908 (PMC4874696; doi:10.1371/journal.pcbi.1004908)
Supplement: S4 Table — Here 3D domains identified by the Arrowhead algorithm were used. (PDF) [file pcbi.1004908.s004.pdf]

Motif ID	TF Name	Consensus	Beta	Motif ID	TF Name	Consensus	Beta	Motif ID	TF Name	Consensus	Beta	Motif ID	TF Name	Consensus	Beta
LM1_RFX1	RFX1	BDGTTKCCATGGMAACMV	0	M00661	NF-muE1	[illegible]	[illegible]	[illegible]	[illegible]	[illegible]	[illegible]	[illegible]	[illegible]	[illegible]	[illegible]
LM2_CTCP	CTCP	NNDCCASHAGRKGGCASYV	[illegible]	M00662	NF-1	[illegible]	[illegible]	[illegible]	[illegible]	[illegible]	[illegible]	[illegible]	[illegible]	[illegible]	[illegible]
[illegible]	[illegible]	[illegible]	[illegible]	[illegible]	PU.1	[illegible]	[illegible]	[illegible]	[illegible]	[illegible]	[illegible]	[illegible]	[illegible]	[illegible]	[illegible]
M00001	MyoD	[illegible]	[illegible]	[illegible]	[illegible]	[illegible]	[illegible]	[illegible]	[illegible]	[illegible]	[illegible]	[illegible]	[illegible]	[illegible]	[illegible]
M00002	E47	[illegible]	0	[illegible]	TCF-4	[illegible]	[illegible]	[illegible]	[illegible]	[illegible]	[illegible]	[illegible]	[illegible]	[illegible]	[illegible]
M00004	c-Myb	[illegible]	[illegible]	[illegible]	TBP	[illegible]	[illegible]	[illegible]	[illegible]	[illegible]	[illegible]	[illegible]	[illegible]	[illegible]	[illegible]
M00005	AP-4	[illegible]	0	[illegible]	[illegible]	[illegible]	[illegible]	[illegible]	[illegible]	[illegible]	[illegible]	[illegible]	[illegible]	[illegible]	[illegible]
M00007	Elk-1	[illegible]	[illegible]	[illegible]	alpha-CP1	[illegible]	[illegible]	[illegible]	[illegible]	[illegible]	[illegible]	[illegible]	[illegible]	[illegible]	[illegible]
M00011	Evi-1	[illegible]	0	[illegible]	ATF1	[illegible]	[illegible]	[illegible]	[illegible]	[illegible]	[illegible]	[illegible]	[illegible]	[illegible]	[illegible]
M00017	ATF	[illegible]	[illegible]	[illegible]	E12	[illegible]	[illegible]	[illegible]	[illegible]	[illegible]	[illegible]	[illegible]	[illegible]	[illegible]	[illegible]
M00025	Elk-1	[illegible]	[illegible]	[illegible]	[illegible]	[illegible]	[illegible]	[illegible]	[illegible]	[illegible]	[illegible]	[illegible]	[illegible]	[illegible]	[illegible]
M00026	RSRFC4	[illegible]	0	[illegible]	HEB	[illegible]	[illegible]	[illegible]	[illegible]	[illegible]	[illegible]	[illegible]	[illegible]	[illegible]	[illegible]
M00032	[illegible]	[illegible]	[illegible]	[illegible]	[illegible]	[illegible]	[illegible]	[illegible]	[illegible]	[illegible]	[illegible]	[illegible]	[illegible]	[illegible]	[illegible]
M00037	NF-E2	[illegible]	[illegible]	[illegible]	SMAD3	[illegible]	[illegible]	[illegible]	[illegible]	[illegible]	[illegible]	[illegible]	[illegible]	[illegible]	[illegible]
M00039	CREB	[illegible]	0	[illegible]	CACCC-binding factor	[illegible]	[illegible]	[illegible]	[illegible]	[illegible]	[illegible]	[illegible]	[illegible]	[illegible]	[illegible]
M00040	ATF2	[illegible]	[illegible]	[illegible]	SP1	[illegible]	[illegible]	[illegible]	[illegible]	[illegible]	[illegible]	[illegible]	[illegible]	[illegible]	[illegible]
M00041	ATF2:c-Jun	[illegible]	[illegible]	[illegible]	Cdx-2	[illegible]	[illegible]	[illegible]	[illegible]	[illegible]	[illegible]	[illegible]	[illegible]	[illegible]	[illegible]
[illegible]	[illegible]	[illegible]	[illegible]	[illegible]	SMAD4	[illegible]	[illegible]	[illegible]	[illegible]	[illegible]	[illegible]	[illegible]	[illegible]	[illegible]	[illegible]
M00050	E2F	[illegible]	[illegible]	[illegible]	E2F-1:DP-1	[illegible]	[illegible]	[illegible]	[illegible]	[illegible]	[illegible]	[illegible]	[illegible]	[illegible]	[illegible]
M00051	NF-kappaB (p50)	[illegible]	[illegible]	[illegible]	E2F-1:DP-2	[illegible]	[illegible]	[illegible]	[illegible]	[illegible]	[illegible]	[illegible]	[illegible]	[illegible]	[illegible]
M00052	NF-kappaB (p65)	[illegible]	[illegible]	[illegible]	E2F-4:DP-1	[illegible]	[illegible]	[illegible]	[illegible]	[illegible]	[illegible]	[illegible]	[illegible]	[illegible]	[illegible]
M00053	c-Rel	[illegible]	[illegible]	[illegible]	E2F-4:DP-2	[illegible]	[illegible]	[illegible]	[illegible]	[illegible]	[illegible]	[illegible]	[illegible]	[illegible]	[illegible]
M00054	NF-kappaB	[illegible]	[illegible]	[illegible]	[illegible]	[illegible]	[illegible]	[illegible]	[illegible]	[illegible]	[illegible]	[illegible]	[illegible]	[illegible]	[illegible]
M00055	N-Myc	[illegible]	0	[illegible]	EF-1	[illegible]	[illegible]	[illegible]	[illegible]	[illegible]	[illegible]	[illegible]	[illegible]	[illegible]	[illegible]
M00058	HEN1	[illegible]	0	[illegible]	PPAR,HNF-4,COUP,RAR	[illegible]	[illegible]	[illegible]	[illegible]	[illegible]	[illegible]	[illegible]	[illegible]	[illegible]	[illegible]
M00062	IRF-1	[illegible]	0	[illegible]	PPAR direct repeat 1	[illegible]	[illegible]	[illegible]	[illegible]	[illegible]	[illegible]	[illegible]	[illegible]	[illegible]	[illegible]
[illegible]	[illegible]	[illegible]	[illegible]	[illegible]	[illegible]	[illegible]	[illegible]	[illegible]	[illegible]	[illegible]	[illegible]	[illegible]	[illegible]	[illegible]	[illegible]
