# Supplementary figures and images for: Computational Identification of Genomic Features That Influence 3D Chromatin Domain Formation
Source: PLoS Comput Biol. 2016 May 20;12(5):e1004908. doi: 10.1371/journal.pcbi.1004908 (PMC4874696; doi:10.1371/journal.pcbi.1004908)

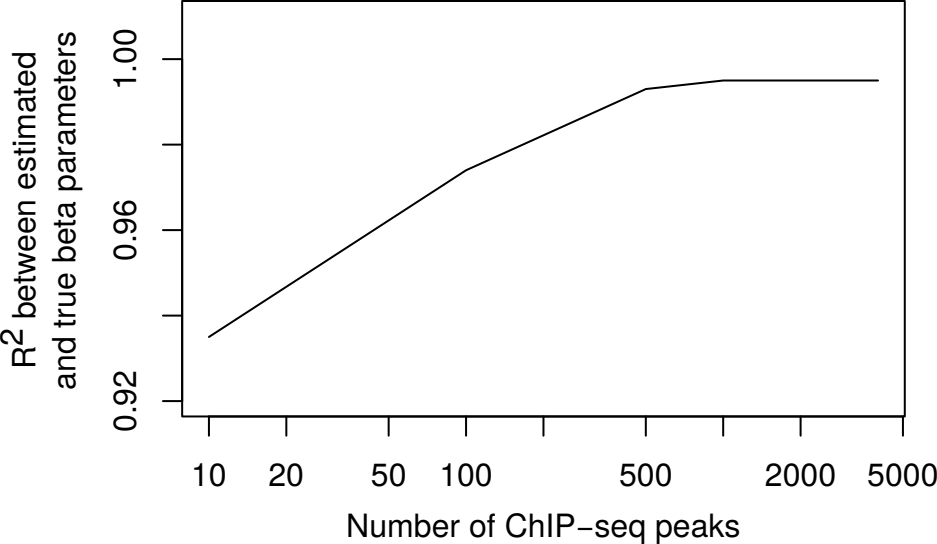

Supplement: S1 Fig — (PDF) [file pcbi.1004908.s005.pdf]

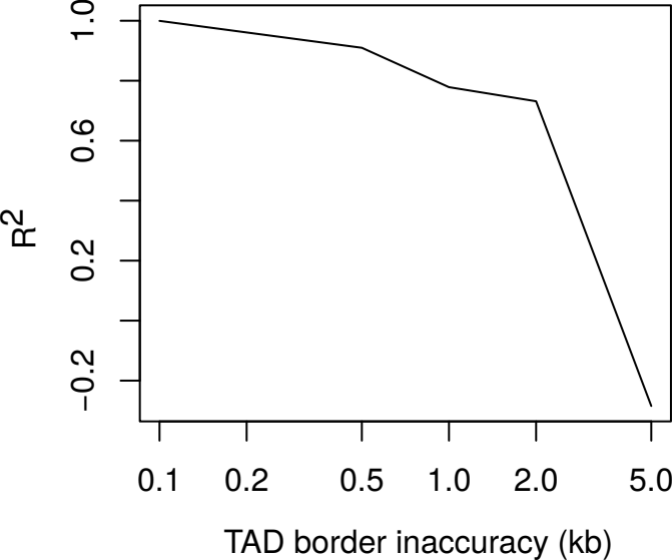

Supplement: S2 Fig — R squared is computed between beta parameters estimated from TAD borders and beta parameters estimated from TAD borders with random noise. Random noise was drawn from a normal distribution of mean zero and varying standard deviations in kb (x-axis). (PDF) [file pcbi.1004908.s006.pdf]

Enrichment Test

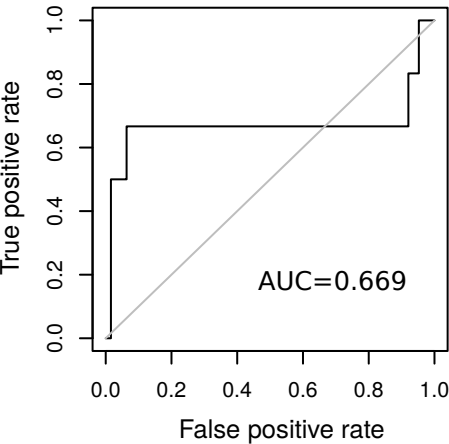

Random Forests

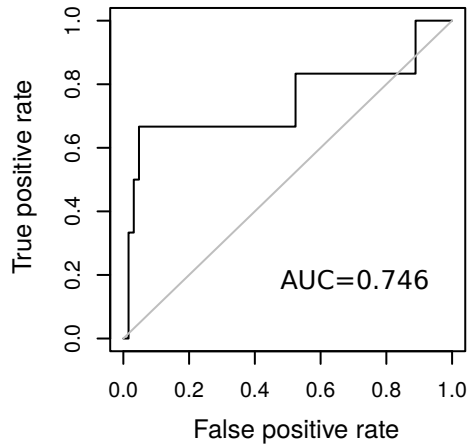

Multiple Logistic Regression

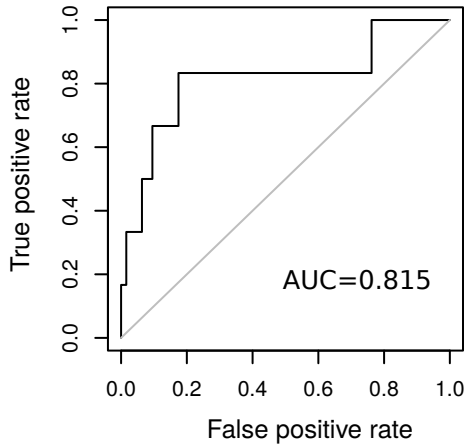

Supplement: S3 Fig — Receiver operating characteristic (ROC) curves were computed from Wald’s statistics for ET, beta parameters for MLR, and variable importances for random forests. (PDF) [file pcbi.1004908.s007.pdf]

Enrichment test

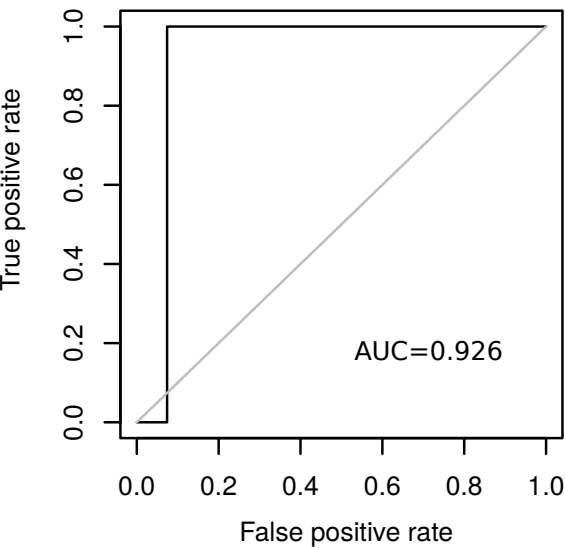

Random forests

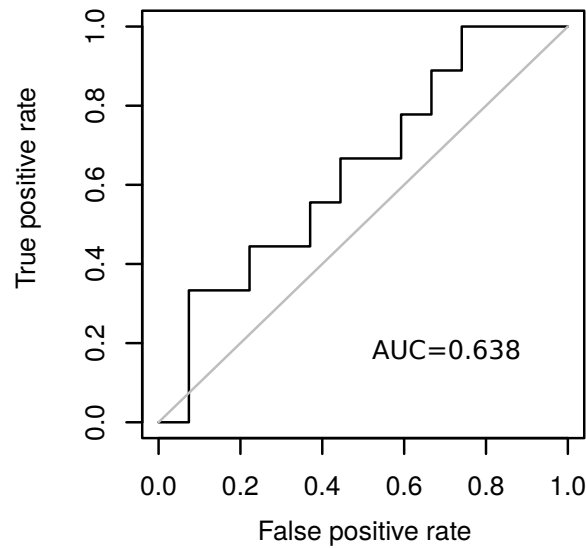

Multiple logistic regression

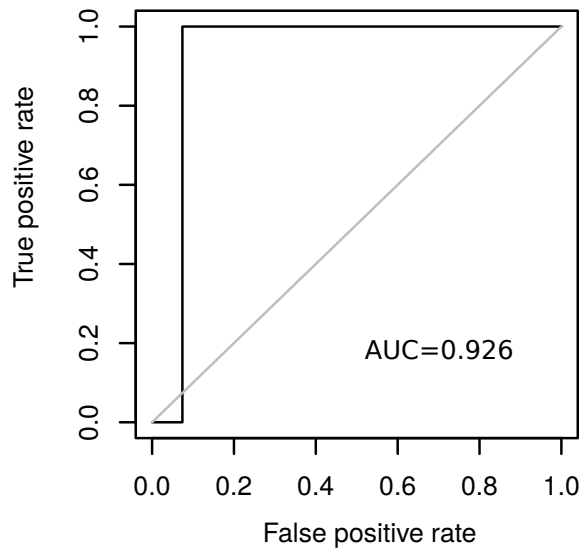

Supplement: S4 Fig — Receiver operating characteristic (ROC) curves were computed from Wald’s statistics for ET, from beta parameters for MLR, and from variable importances for random forests. Computations were carried out at 1 kb resolution. (PDF) [file pcbi.1004908.s008.pdf]

# BEAF-32 consensus motif: CGATA

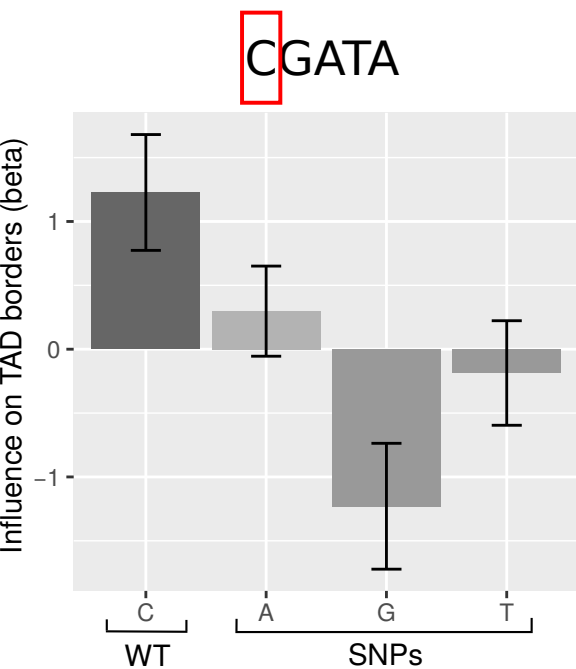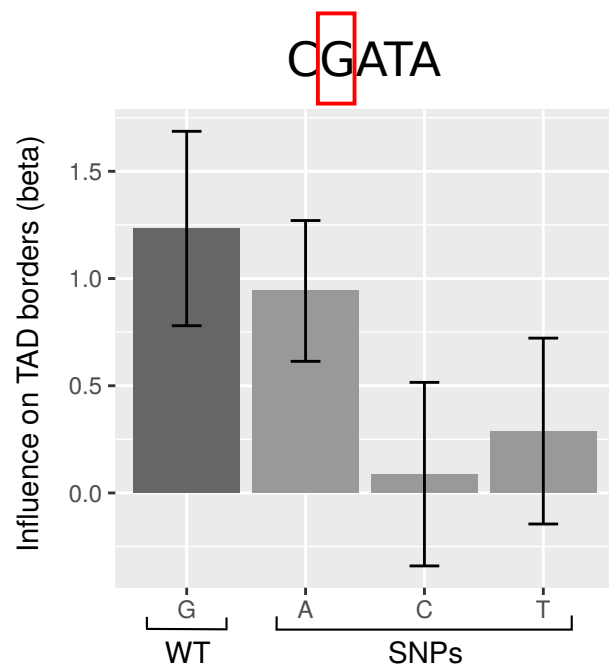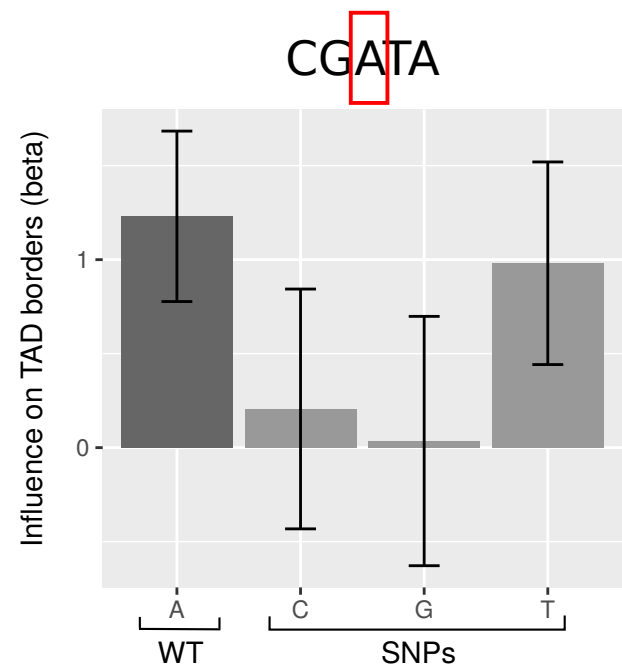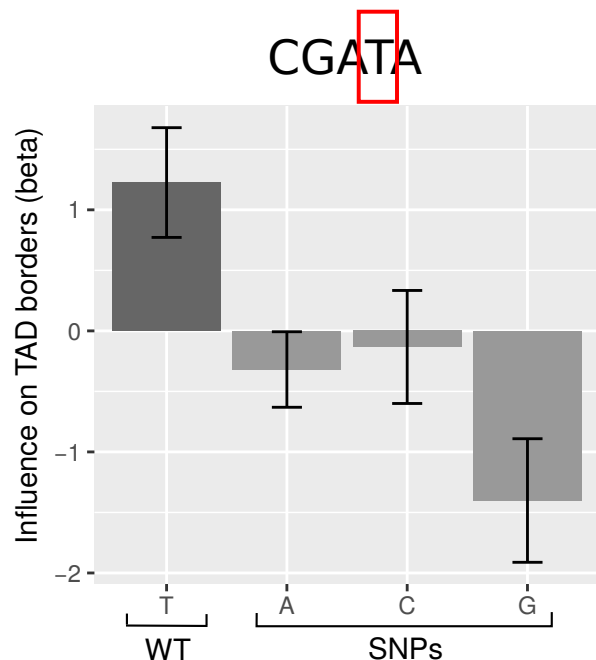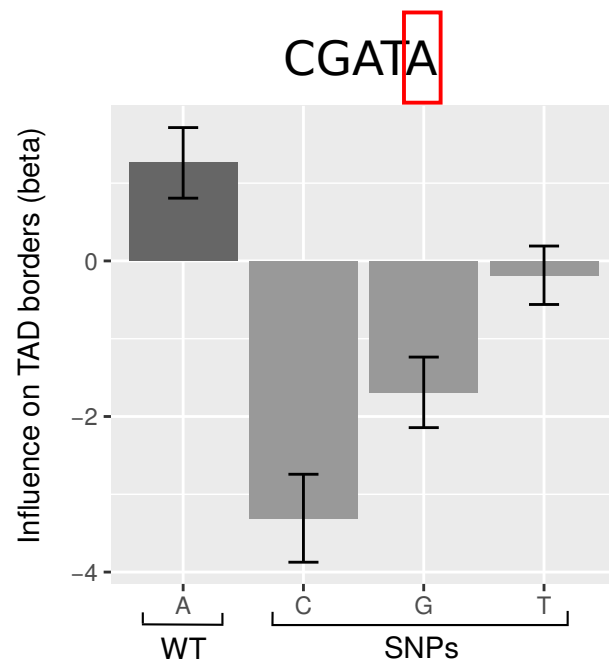

Supplement: S5 Fig — (PDF) [file pcbi.1004908.s009.pdf]

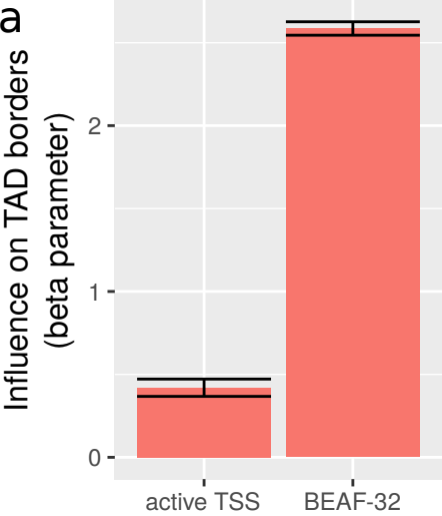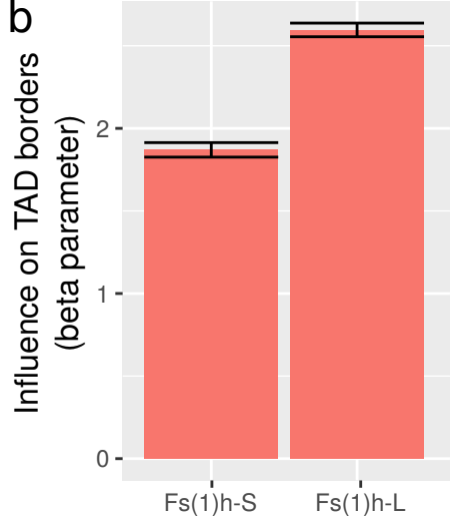

Supplement: S6 Fig — a) Multiple logistic regression of active TSSs and BEAF-32. b) Multiple logistic regression of Fs(1)h-S and Fs(1)h-L. (PDF) [file pcbi.1004908.s010.pdf]

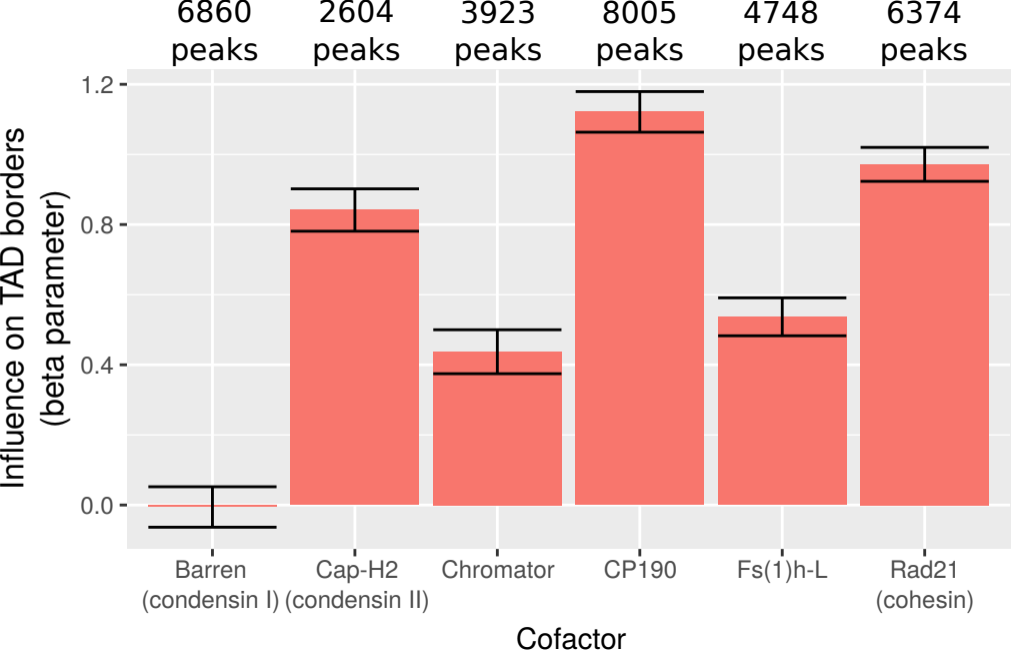

Supplement: S7 Fig — (PDF) [file pcbi.1004908.s011.pdf]
